# Eye-specific retinogeniculate segregation proceeds normally following disruption of patterned spontaneous retinal activity

**DOI:** 10.1186/1749-8104-9-25

**Published:** 2014-11-07

**Authors:** Colenso M Speer, Chao Sun, Lauren C Liets, Ben K Stafford, Barbara Chapman, Hwai-Jong Cheng

**Affiliations:** Center for Neuroscience, University of California, Davis, 1544 Newton Court, Davis, CA 95618 USA; Department of Chemistry and Chemical Biology, Harvard University, 12 Oxford Street, Cambridge, MA 02138 USA; Department of Neurobiology, Physiology, and Behavior, University of California, Davis, One Shields Avenue, Davis, CA 95616 USA; Department of Neurosciences, University of California San Diego, 9500 Gilman Drive, San Diego, CA 92093 USA

**Keywords:** Retinogeniculate, Eye-specific segregation, Retinal wave, Spontaneous activity, Retinal ganglion cell, Dorsal lateral geniculate nucleus

## Abstract

**Background:**

Spontaneous retinal activity (SRA) is important during eye-specific segregation within the dorsal lateral geniculate nucleus (dLGN), but the feature(s) of activity critical for retinogeniculate refinement are controversial. Pharmacologically or genetically manipulating cholinergic signaling during SRA perturbs correlated retinal ganglion cell (RGC) spiking and disrupts eye-specific retinofugal refinement *in vivo*, consistent with an instructive role for SRA during visual system development. Paradoxically, ablating the starburst amacrine cells (SACs) that generate cholinergic spontaneous activity disrupts correlated RGC firing without impacting retinal activity levels or eye-specific segregation in the dLGN. Such experiments suggest that patterned SRA during retinal waves is not critical for eye-specific refinement and instead, normal activity levels are permissive for retinogeniculate development. Here we revisit the effects of ablating the cholinergic network during eye-specific segregation and show that SAC ablation disrupts, but does not eliminate, retinal waves with no concomitant impact on normal eye-specific segregation in the dLGN.

**Results:**

We induced SAC ablation in postnatal ferret pups beginning at birth by intraocular injection of a novel immunotoxin selective for the ferret vesicular acetylcholine transporter (Ferret VAChT-Sap). Through dual-patch whole-cell and multi-electrode array recording we found that SAC ablation altered SRA patterns and led to significantly smaller retinal waves compared with controls. Despite these defects, eye-specific segregation was normal. Further, interocular competition for target territory in the dLGN proceeded in cases where SAC ablation was asymmetric in the two eyes.

**Conclusions:**

Our data demonstrate normal eye-specific retinogeniculate development despite significant abnormalities in patterned SRA. Comparing our current results with earlier studies suggests that defects in retinal wave size, absolute levels of SRA, correlations between RGC pairs, RGC burst frequency, high frequency RGC firing during bursts, and the number of spikes per RGC burst are each uncorrelated with abnormalities in eye-specific segregation in the dLGN. An increase in the fraction of asynchronous spikes occurring outside of bursts and waves correlates with eye-specific segregation defects in studies reported to date. These findings highlight the relative importance of different features of SRA while providing additional constraints for computational models of Hebbian plasticity mechanisms in the developing visual system.

**Electronic supplementary material:**

The online version of this article (doi:10.1186/1749-8104-9-25) contains supplementary material, which is available to authorized users.

## Background

Retinogeniculate development is a classical model for exploring the mechanisms that regulate circuit refinement in the mammalian brain. At birth in mouse and ferret, binocular inputs to the dorsal lateral geniculate nucleus (dLGN) overlap before remodeling to occupy eye-specific territories [1–4]. During refinement, cholinergic starburst amacrine cells (SACs) initiate waves of propagating excitation [5, 6] that drive neighboring retinal ganglion cells (RGCs) to fire spatiotemporally correlated bursts of action potentials [7–10]. The precise pattern of correlated RGC output has been hypothesized to play critical roles in visual circuit development by instructing the remodeling of retinofugal axon terminals in a retinotopic and eye-specific fashion (for a recent review discussing the role of spontaneous activity in circuit development see Kirkby *et al.*[11]).

Consistent with this hypothesis, pharmacological disruptions of cholinergic spontaneous retinal activity lead to abnormal retinogeniculate [12–17], retinocollicular [17–19], and geniculocortical [20, 21] visual pathway development. Similarly, genetic deletion of the ß2 subunit of the nicotinic acetylcholine receptor (ß2(KO)) disrupts spontaneous retinal activity [22–26] and perturbs retinogeniculate [15, 17, 23, 27–29], retinocollicular [17–19, 30–33], and visual geniculocortical development [20, 33–35].

While such experiments demonstrate that some aspect of spontaneous retinal activity driven by SACs is important for eye-specific retinofugal refinement (but, see Cook *et al.*[36]), identifying the feature(s) of activity critical for normal development is controversial. Manipulations of cholinergic signaling disrupt multiple aspects of spontaneous retinal activity, making a systematic investigation of each component extremely difficult [37]. Early experiments using pharmacological tools to increase retinal activity in one or both eyes documented competitive interactions between eye-specific retinogeniculate projections and suggested that retinal waves instruct the formation of eye-specific layers via spatiotemporal patterns of correlated output amongst neighboring RGCs [12, 38].

Subsequent SAC ablation studies from our laboratory suggested the opposite - that while the presence of normal levels of activity was important for development, the normal pattern of RGC activity was not critical for eye-specific segregation [14]. This controversy has been extended in recent years, with experiments concluding that ‘retinal waves are neither necessary nor sufficient for the formation of segregated retinogeniculate projections’ [25] and others emphasizing that ‘the precise spatiotemporal pattern of spontaneous retinal activity instructs neural circuit development’ [39]. This latter study specifically argued that retinal wave size is the critical feature of patterned spontaneous retinal activity necessary for normal eye-specific retinogeniculate refinement, and that smaller waves lead to abnormalities in segregation [39].

In an effort to understand which feature(s) of cholinergic retinal waves are important for eye-specific retinogeniculate development, we ablated SACs in postnatal ferret retinae *in vivo* using a novel Saporin-based immunotoxin selective for ferret SACs (Ferret-specific vesicular acetylcholine transport protein-Saporin (Ferret VAChT-Sap)). Symmetric SAC ablation in the two eyes disrupted spontaneous retinal activity, but did not impact eye-specific segregation, while asymmetric SAC ablation caused the eye with the greater number of SACs to gain target territory at the expense of the other eye. Dual-patch clamp and multi-electrode array (MEA) recordings of neighboring RGCs revealed a loss of correlated spiking between pairs in Ferret VAChT-Sap-treated retinae. Following SAC ablation, retinal waves persisted but were significantly smaller than normal, and RGC burst properties were perturbed. SAC ablation did not impact overall levels of spontaneous retinal activity.

Comparison of these results with previously published data from several different manipulations of spontaneous retinal activity reveals that an increase in the fraction of isolated RGC spiking activity occurring outside burst and wave activity in either eye may be the critical feature of abnormal spontaneous retinal activity correlated with defects in eye-specific segregation. In combination, our data suggest that several features of spontaneous retinal activity appear flexible, or perhaps dispensable, for normal eye-specific retinogeniculate development, and offer new constraints for models of activity-dependent refinement in the developing visual pathways.

## Results

### Eye-specific retinogeniculate projections are segregated after Ferret VAChT-Sap treatment

**Figure 1:**
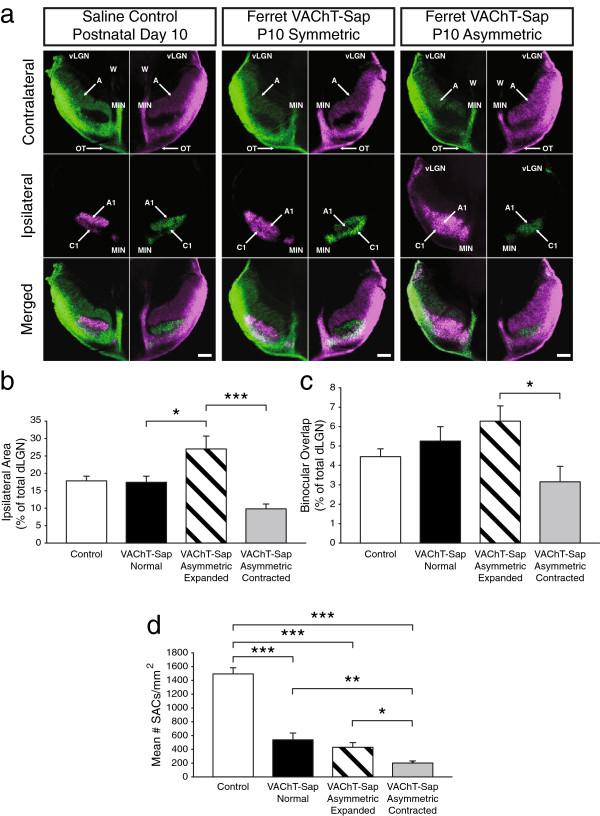
**Effects of Ferret VAChT-Sap treatment on retinogeniculate refinement.** At postnatal day 10 (P10), retinal afferents from the left eye (green in all images) and right eye (magenta) have undergone extensive remodeling to form contralateral layers A and ipsilateral layers A1 (**a**, left panels) segregated from one another in the two dLGNs (**a**, left ‘Merged’ panels). Treatment with Ferret VAChT-Sap does not affect the normal refinement process in cases where the degree of SAC ablation is symmetric in the two eyes (**a**, center panels). Binocular overlap and the overall size of the ipsilateral projection to both dLGNs is normal following Ferret VAChT-Sap treatment when the ablation is symmetric in the two eyes **(b,** **c)**. In cases where treatment with Ferret VAChT-Sap leads to asymmetric SAC ablation in one eye versus the other, the eye with the greater number of SACs develops an expanded projection in the two dLGNs (**a**, right panels; **b**, **c**, quantification). Binocular overlap is greater in the dLGN receiving the expanded ipsilateral projection compared with the dLGN receiving the contracted ipsilateral projection **(c)**. In all cases of Ferret VAChT-Sap treatment, the number of SACs is significantly reduced compared to saline controls **(d)**. C1, ipsilateral layer; dLGN, dorsal lateral geniculate nucleus; MIN, medial intralaminar nucleus; OT, optic tract; SAC, starburst amacrine cells; vLGN, ventral lateral geniculate nucleus; W, wing of the geniculate. Quantification shows mean values + SEM; two saline controls, four symmetric Ferret VAChT-Sap-treated animals, and ten asymmetric VAChT-Sap-treated animals were included in the analysis. Statistics reflect a one-way ANOVA with Bonferroni *post-hoc* correction (*P <0.05, **P <0.01, ***P <0.001). All group mean comparisons with no corresponding asterisks did not reach significance.

The SAC network drives patterned wave activity during the first postnatal week of development in the ferret [6, 12, 40], making this population of interneurons a prime target for loss-of-function ablation studies. To evaluate the role of cholinergic spontaneous retinal activity in the refinement of eye-specific projections to the dLGN, we ablated SACs beginning at birth and examined retinogeniculate central anatomy at postnatal day 10 (P10) when eye-specific segregation in the ferret dLGN is largely complete (Figure 1) [3, 4]. In saline controls at P10, retinogeniculate afferents from each eye occupy non-overlapping domains in the dLGN (Figure 1a, left panels). Binocular overlap at P10 was 4.48 ± 0.39% (mean ± standard error of mean (SEM) of the total dLGN area in saline controls, similar to normal ferrets at this age (Figure 1c) [4].

In animals treated with Ferret VAChT-Sap, the degree of SAC ablation was dose-dependent (Additional file 1). For all experiments we elected to use the dosage yielding maximal SAC ablation (3.52 μg/eye). Occasional interocular variations in the effects of Ferret VAChT-Sap eye-injections resulted in distinct phenotypes correlating with the degree to which SAC ablation was symmetric in the two eyes. In animals where SAC ablation was symmetrical in the two eyes, eye-specific projections to the dLGN at P10 were normal relative to saline controls (Figure 1a, center panels). The percentage of dLGN area occupied by ipsilateral projections in symmetric Ferret VAChT-Sap cases (17.76 ± 1.45%; mean ± SEM) was not significantly different from that in saline controls (18.09 ± 1.29%; mean ± SEM) (Figure 1b), and the overall size of the dLGN was not impacted by SAC ablation (data not shown). Eye-specific segregation was normal and binocular overlap in symmetric Ferret VAChT-Sap cases (5.30 ± 0.71% of the total dLGN area; mean ± SEM) was not significantly different from saline controls (Figure 1c). In Ferret VAChT-Sap-treated animals with normal retinogeniculate projections the mean density of SACs was 550.73 ± 94.85 cells/mm^2^ (mean ± SEM) compared to 1512 ± 70.37 cells/mm^2^ (mean ± SEM) in saline controls, a 63.58% reduction (Figure 1d). The representative normal development of retinogeniculate afferents presented in Figure 1a was from a case exhibiting a mean 78.22% SAC ablation in each eye (center panels).By contrast, animals exhibiting asymmetric effects of Ferret VAChT-Sap treatment in the two eyes had a different pattern of retinogeniculate projections than those of saline controls or symmetric Ferret VAChT-Sap-treated cases. Asymmetric SAC ablation resulted in a shift in eye-specific competition for target territory in the dLGN. On one side of the brain the ipsilateral retinogeniculate layer had expanded to occupy a larger fraction of the dLGN (27.32 ± 3.42%; mean ± SEM), while on the other side of the brain the ipsilateral projection had contracted to occupy a significantly smaller territory (10.14 ± 1.19%; mean ± SEM) (Figure 1a, right panels; Figure 1b, quantification).

The dLGN containing the expanded ipsilateral projections showed greater binocular overlap (6.32 ± 0.75% of the total dLGN area; mean ± SEM) than that measured in the opposite dLGN containing the contracted ipsilateral projections (3.20 ± 0.78% of the total dLGN area; mean ± SEM) (Figure 1c). Interestingly, in such cases of asymmetric SAC ablation, the ‘winning’ eye with expanded retinogeniculate projections averaged 445.90 ± 61.75 SACs/mm^2^ (mean ± SEM), a 70.51% ablation relative to saline controls (Figure 1d). The ‘losing’ eye with contracted retinogeniculate projections averaged 215.13 ± 22.24 SACs/mm^2^ (mean ± SEM), a 51.75% reduction compared to the ‘winning’ eye, and an 85.77% ablation compared to saline controls (Figure 1d).

The competitive interactions observed in cases of asymmetric SAC ablation were not likely due to non-specific effects of Ferret VAChT-Sap on cell populations other than SACs, since immunotoxin treatment did not cause RGC ablation in our experiments (Additional files 1, 2, 3 and 4). The effects are consistent with residual spontaneous retinal activity driving competition for target territory in the dLGN whenever there are activity imbalances between the two eyes. To test this hypothesis and determine the effects of SAC ablation on spontaneous retinal activity, we recorded spontaneous activity patterns in control and Ferret VAChT-Sap-treated retinae using both dual whole-cell paired patch and multi-electrode recording techniques.

### Dual whole-cell patch recording following Ferret VAChT-Sap treatment reveals a decrease in correlated activity between neighboring retinal ganglion cells with no impact on overall firing frequency

**Figure 2:**
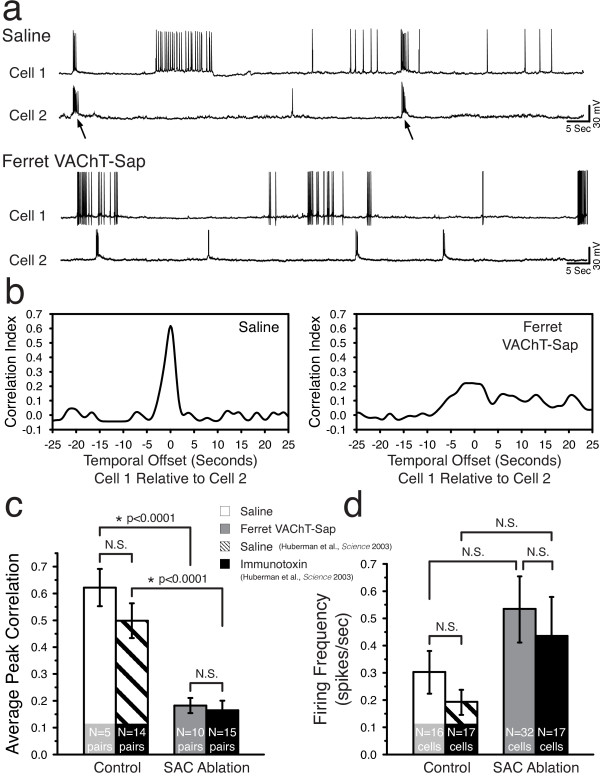
**Starburst amacrine cell ablation disrupts correlated spiking between neighboring retinal ganglion cells.** Neighboring RGCs in saline control retinae exhibit correlated bursts of action potentials (**a**, arrows in top traces). Treatment with Ferret VAChT-Sap disrupts correlated firing between neighboring RGC pairs (**a**, bottom traces). Quantification of the traces in **(a)** reveals a sharp peak in the cross-correlation function for the saline control pair (**b**, left panel) which is absent in the Ferret VAChT-Sap-treated RGC pair (**b**, right panel). This disruption of peak correlation index values following Ferret VAChT-Sap treatment is significant for the entire population of recorded pairs (**c**, saline in white bar and Ferret VAChT-Sap in grey bar). The values recorded in the current study are not significantly different from those previously reported using a different immunotoxin (**c**, saline in black striped bar and immunotoxin in black bar; data reanalyzed from Huberman *et al.*[14]). Treatment with Ferret VAChT-Sap did not significantly impact overall retinal activity levels relative to saline controls (**d**, saline in white bar and Ferret VAChT-Sap in grey bar), a result reported previously using a different immunotoxin (**d**, saline in black striped bar and immunotoxin in black bar; data reanalyzed from Huberman *et al.*[14]). Quantification shows mean ± SEM; statistics reflect two-tailed *P* values calculated from independent two sample Student’s t-tests. The number of retinae recorded for Ferret VAChT-Sap and saline conditions at P2 was N = 2 and 3, P6 N = 4 and 3, P9 N = 1 and 1, and P10 N = 1 and 1, respectively. NS, not significant.

Previous work from our group explored the effects of SAC ablation on eye-specific segregation in ferrets using a different immunotoxin. In that study, dual whole-cell patch recordings were used to evaluate correlations in spiking between neighboring RGCs under control and experimental conditions [14]. To directly compare our results using Ferret VAChT-Sap to those obtained with the previous immunotoxin, we performed a series of dual whole-cell patch recording experiments and compared our data directly to those obtained in the previous study (see Methods) [14]. Figure 2a depicts representative recording traces from two cell pairs in saline and Ferret VAChT-Sap-treated retina. RGC pairs in control retinae exhibited well-correlated bursts of action potentials or compound excitatory postsynaptic currents (Figure 2a, arrows in top panel). In contrast, RGC pairs in Ferret VAChT-Sap-treated retinae were uncoordinated in their burst and spike behavior (Figure 2a, bottom panel). The cross-correlation function for the pairs shown in Figure 2a revealed a sharp peak for the controls, indicating strong correlation in the activity of the pair (Figure 2b, left panel), while the peak correlation in the Ferret VAChT-Sap pair was significantly diminished (Figure 2b, right panel). Quantifying population data for all pairs confirmed a significant decrease in the peak correlation values of Ferret VAChT-Sap-treated RGC pairs relative to saline controls (Figure 2c). Interestingly, the values we measured for each condition in the current study were not significantly different from those obtained in the previous study using a different immunotoxin, indicating the effect of the manipulation was the same in both cases (Figure 2c).Additional analysis of all recorded RGC spike trains (both pairs and single cells) revealed that Ferret VAChT-Sap treatment did not impact overall firing frequency relative to saline controls (Figure 2d). Again, the values we recorded in the current experiments were not significantly different from those reported using the previous immunotoxin (Figure 2d).

### Multi-electrode array recordings show that Ferret VAChT-Sap treatment leads to smaller retinal waves relative to controls

**Figure 3:**
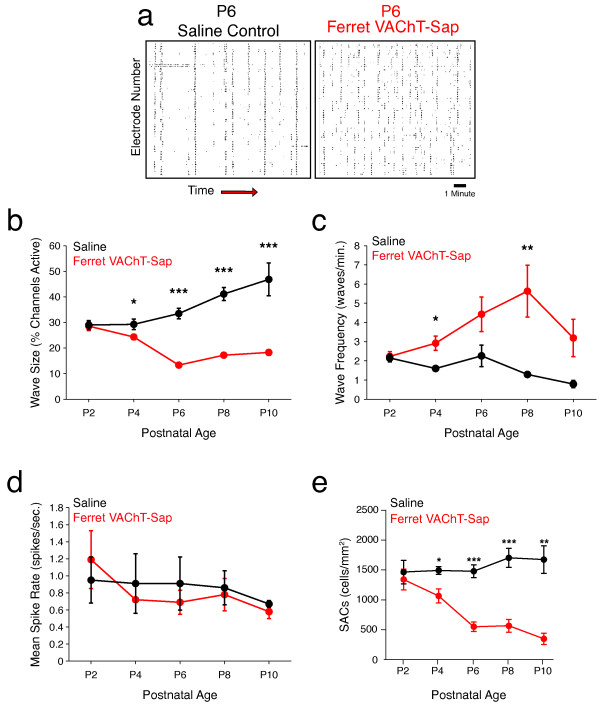
**Ferret VAChT-Sap treatment results in smaller and more frequent wave events concomitant with starburst amacrine cell loss.** In control recordings, such as this example raster of a P6 saline recording, waves pass across the array every one to three minutes, with little spontaneous activity in between wave events (**a**, left panel). In contrast, waves occur more frequently and with smaller domains of propagating activity in Ferret VAChT-Sap-treated retinae (**a** right panel, P6 treated eye from same animal as in **(a)** left panel). Quantification of wave size reveals a significant decrease in the number of electrodes with spike activity per wave event in Ferret VAChT-Sap-treated retinae compared to controls **(b)**. In addition to reducing wave size, treatment with Ferret VAChT-Sap leads to an increase in the number of independent wave events per minute relative to saline control values **(c)**. The mean spike rates between controls and Ferret VAChT-Sap-treated retinae are not significantly different **(d)**. Ferret VAChT-Sap treatment leads to a progressive decline in the density of SACs measured in retinae used for MEA recording over development relative to controls **(e)**. All values represent mean ± SEM; the number of retinae/animals recorded for Ferret VAChT-Sap and Saline conditions at P2 was N = 6 and 5, P4 N = 7 and 4, P6 N = 7 and 6, P8 N = 7 and 6, and P10 N = 4 and 2 respectively. Statistics reflect two-tailed *P* values calculated from independent two-sample Student’s t-tests (*P <0.05, **P <0.01, ***P <0.001).

Cholinergic retinal waves occurring during retinogeniculate refinement generate synchronous bouts of spike activity amongst neighboring RGCs, with a falloff in correlations as a function of distance between RGC pairs [9, 10]. Such longer-range correlational structure is not amenable to study using dual whole-cell patch recordings, and previous experiments relying on this approach would have missed important features of spontaneous retinal activity on this basis [14]. To address this issue, we opted to record from control and Ferret VAChT-Sap-treated retinae using a 64-channel MEA. In saline control recordings, large waves often elicited propagating activity on all channels of the recording array (Figure 3a, left panel) and wave size increased progressively from P2 to P10 (Figure 3b). In Ferret VAChT-Sap-treated retinae waves were present at all developmental ages examined and were evident even in cases where more than 85% of SACs were ablated. This is consistent with the previous study from our lab which reported that calcium waves were initially present in immunotoxin-treated retinae and then disappeared after several days, in some cases persisting until as late as P6 [14]. However, our MEA recordings revealed that retinal waves in Ferret VAChT-Sap-treated retinae were consistently smaller than those measured in controls at P4 and at each time point thereafter (Figure 3b). These small, residual waves may have been driven by the remaining SACs not killed by the immunotoxin, given that individual SACs are capable of initiating wave events [41]. Further, it is unlikely that residual waves were driven by gap-junction-mediated signaling since a reversion to gap-junction-mediated wave activity, such as occurs in mice with deficiencies in cholinergic signaling [25, 26, 42], leads to enlarged waves with increased velocity that were never observed in Ferret VAChT-Sap-treated retinae. Future pharmacological experiments are needed to confirm the source of residual wave activity following SAC ablation.

### Multi-electrode array recordings show that Ferret VAChT-Sap treatment increases retinal wave frequency

In addition to decreasing the size of retinal waves, treatment with Ferret VAChT-Sap increased the frequency of wave activity relative to controls (Figure 3c). In saline control recordings, retinal wave frequency decreased from P2 to P10 (Figure 3c, Table 1). In contrast, retinal wave frequency was consistently higher in Ferret VAChT-Sap-treated retinae at all time points except P2 (Figure 3c, Table 1). Averaged over the entire epoch of eye-specific segregation, retinal waves in saline control animals occurred 1.62 ± 0.15 times each minute (P2 to P10 grouped mean ± SEM, data not shown) while waves in Ferret VAChT-Sap-treated retinae occurred 3.76 ± 0.42 times each minute (P2 to P10 grouped mean ± SEM, data not shown).

**Table 1:** **RGC burst and wave properties recorded across development in control and Ferret VAChT-Sap-treated retinae**

All Bursts	P2 Saline	P2 ITOX	P4 Saline	P4 ITOX	P6 Saline	P6 ITOX	P8 Saline	P8 ITOX	P10 Saline	P10 ITOX
Total Neurons	257	316	247	418	463	355	334	245	175	184
Total Neurons Analyzed for Bursts	255	316	244	415	457	336	328	241	175	179
Bursts Per Neuron	10.04 ± 1.21	12.34 ± 1.42	11.47 ± 3.28	14.98 ± 4.81	10.37 ± 2.16	12.36 ± 1.22	8.36 ± 0.76	16.88 ± 4.33*	7.66 ± 1.40	16.23 ± 4.27
Burst Frequency (bursts/min)	0.67 ± 0.08	0.82 ± 0.09	0.76 ± 0.22	1.00 ± .32	0.69 ± 0.14	0.82 ± 0.08	0.55 ± 0.05	1.12 ± 0.29*	0.51 ± 0.09	1.08 ± 0.28
% Spikes in Bursts	99.14 ± 0.10	98.13 ± 0.55	97.67 ± 0.79	96.49 ± 1.82	98.18 ± 0.61	95.28 ± 1.60	97.96 ± 0.65	94.69 ± 1.19*	96.04 ± 1.58	91.50 ± 2.54
Maximum Burst Spike Rate	459.76 ± 63.76	473.06 ± 162.98	755.15 ± 187.18	467.56 ± 155.52	528.04 ± 85.41	528.48 ± 161.02	510.52 ± 62.51	438.55 ± 199.77	643.55 ± 115.61	623.60 ± 292.19
% of Total Burst Time >10 Hz	58.97 ± 4.10	57.51 ± 4.54	57.03 ± 4.44	53.48 ± 3.53	61.42 ± 3.60	42.82 ± 3.54***	67.42 ± 2.8	40.14 ± 4.43***	64.59 ± 0.53	33.53 ± 4.52***
% Bursts in Waves	87.77 ± 2.76	80.62 ± 6.6	83.95 ± 2.48	77.20 ± 4.69	86.74 ± 3.20	76.34 ± 5.60	90.96 ± 3.43	80.73 ± 3.33*	91.27 ± 1.51	72.65 ± 7.34*
Mean Burst Spike Rate (spikes/s)	34.75 ± 4.43	44.85 ± 8.02	70.56 ± 22.46	42.67 ± 9.09	41.08 ± 7.12	27.84 ± 10.20	51.13 ± 7.56	33.38 ± 12.41	49.12 ± 10.56	53.08 ± 36.94
Burst Interspike Interval (ISI) (ms)	64.1 ± 5.44	76.66 ± 10.7	91.99 ± 19.16	96.57 ± 15.53	72.20 ± 11.62	132.11 ± 23.90*	63.42 ± 13.40	140.63 ± 27.85**	64.22 ± 10.16	189.79 ± 26.99***
Mean Spike Rate (spikes/s)	0.95 ± 0.27	1.19 ± 0.34	0.91 ± 0.35	0.72 ± .16	0.91 ± 0.31	0.69 ± 0.14	0.86 ± 0.20	0.78 ± 0.19	0.67 ± 0.04	0.58 ± 0.08
Burst Duration (s)	2.01 ± 0.18	1.93 ± 0.27	2.20 ± 0.27	1.82 ± 0.16	1.94 ± 0.23	2.25 ± 0.19	2.08 ± 0.14	2.10 ± 0.35	2.19 ± 0.41	2.30 ± 0.39
	**P2 Saline**	**P2 ITOX**	**P4 Saline**	**P4 ITOX**	**P6 Saline**	**P6 ITOX**	**P8 Saline**	**P8 ITOX**	**P10 Saline**	**P10 ITOX**
Wave Bursts										
Burst Duration	1.96 ± 0.11	1.91 ± 0.22	2.07 ± 0.42	1.85 ± 0.16	1.93 ± 0.23	2.14 ± 0.20	2.14 ± 0.18	2.10 ± 0.23	2.30 ± 0.52	2.52 ± 0.24
Burst Spike Rate	32.47 ± 6.22	40.18 ± 7.08	56.85 ± 18.58	36.04 ± 8.14	37.14 ± 5.34	21.63 ± 2.60*	46.52 ± 5.34	25.38 ± 7.12*	51.40 ± 12.62	40.06 ± 25.54
Burst ISI	62.59 ± 5.72	70.74 ± 8.87	70.78 ± 16.25	90.59 ± 12.28	67.71 ± 9.09	131.06 ± 21.76**	58.05 ± 9.48	132.27 ± 23.50**	52.65 ± 3.44	176.17 ± 20.79***
Burst Spike Rate from ISI	16.26 ± 1.37	14.61 ± 1.69	15.02 ± 2.90	12.01 ± 1.78	15.98 ± 2.24	8.11 ± 1.33**	18.38 ± 2.24	8.13 ± 1.46***	19.03 ± 1.24	5.79 ± 0.72***
Interburst Interval (IBI)	144.15 ± 18.28	126.84 ± 17.00	155.55 ± 35.60	167.87 ± 27.30	154.43 ± 27.59	143.91 ± 10.34	164.20 ± 10.60	113.82 ± 36.40	184.21 ± 6.98	161.42 ± 49.09
Maximum Burst Spike Rate	489.97 ± 73.78	480.11 ± 127.77	674.90 ± 174.07	430.41 ± 162.99	529.49 ± 66.71	456.35 ± 140.18	504.49 ± 98.33	395.51 ± 189.97	634.39 ± 62.69	563.02 ± 291.44
% Neurons Bursting Per Second In Waves	4.01 ± 0.41	4.52 ± 0.47	4.84 ± 0.30	4.14 ± 0.46	5.01 ± 0.35	3.04 ± 0.15***	5.24 ± 0.42	3.71 ± 0.48*	8.80 ± 0.93	3.48 ± 0.26***
Bursts Per Neuron Per Wave	1.04 ± 0.02	1.05 ± 0.03	1.10 ± 0.07	1.06 ± 0.06	1.12 ± 0.07	1.17 ± 0.14	1.21 ± 0.10	1.04 ± 0.02	1.45 ± 0.25	1.04 ± 0.01
Percent Burst Time >10 Hz	59.71 ± 3.32	57.97 ± 4.15	59.14 ± 5.44	53.51 ± 3.54	60.17 ± 3.60	42.37 ± 3.22***	66.71 ± 3.15	39.72 ± 4.91***	66.80 ± 0.50	33.53 ± 4.69***
Non-Wave Bursts										
Burst Duration	2.05 ± 0.30	1.99 ± 0.34	2.23 ± 0.44	1.83 ± 0.18	1.99 ± 0.24	2.34 ± 0.20	1.97 ± 0.16	2.09 ± 0.46	1.94 ± 0.08	2.14 ± 0.49
Burst Spike Rate	34.96 ± 3.72	49.24 ± 10.32	115.04 ± 45.04	50.14 ± 13.36	46.14 ± 10.29	31.83 ± 17.60	59.78 ± 11.62	42.19 ± 21.54	28.96 ± 9.93	60.44 ± 40.62
Burst ISI	65.37 ± 10.46	77.49 ± 14.19	81.52 ± 29.22	103.91 ± 19.46	77.88 ± 14.38	144.92 ± 32.94*	70.38 ± 21.99	147.73 ± 33.29*	101.08 ± 55.77	207.30 ± 20.09
Burst Spike Rate from ISI	34.96 ± 3.72	49.24 ± 10.32	115.04 ± 45.04	50.14 ± 13.36	46.14 ± 29	31.83 ± 17.60	59.78 ± 11.62	42.19 ± 21.54	28.96 ± 9.93	60.44 ± 40.62
Maximum Burst Spike Rate	437.86 ± 100.60	479.49 ± 209.59	1016.96 ± 333.70	477.51 ± 143.01	496.95 ± 117.20	583.86 ± 188.27	607.25 ± 47.91	477.49 ± 194.47	624.04 ± 310.54	751.96 ± 354.12
Percent Burst Time >10 Hz	57.56 ± 4.76	56.82 ± 4.32	56.22 ± 4.62	52.17 ± 4.42	59.43 ± 4.75	40.89 ± 3.93**	65.61 ± 3.19	39.29 ± 4.67***	51.51 ± 12.81	32.16 ± 3.04
Wave size (% of channels)	29.01 ± 1.77	28.55 ± 1.69	29.26 ± 2.07	24.32 ± 0.98*	33.48 ± 2.03	13.33 ± 0.50***	41.11 ± 2.56	17.21 ± 0.57***	46.83 ± 6.44	18.28 ± 1.14***
Wave frequency (waves/min)	2.15 ± 0.20	2.23 ± 0.25	1.60 ± 0.12	2.92 ± 0.37*	2.26 ± 0.56	4.43 ± 0.90	1.29 ± 0.13	5.63 ± 1.35**	0.79 ± 0.19	3.19 ± 0.97

The table list mean values ± SEM for all parameters recorded. Statistics reflect two-tailed *P* values calculated from independent two-sample Student’s t-tests (*P <0.05, **P <0.01, ***P <0.001). ITOX, Ferret VAChT-Sap-treated; P2-P10, postnatal days 2–10; ISI, interspike interval; IBI, interburst interval.

In light of the increased frequency of retinal waves induced by SAC ablation, we evaluated whether overall activity levels were increased in immunotoxin-treated versus saline control retinae. The mean spike rate for all recorded RGCs was not significantly different in Ferret VAChT-Sap-treated retinae compared to saline controls at any time points investigated (Figure 3d, Table 1). This confirms the results from our dual whole-cell patch experiments and demonstrates that SAC ablation does not impact overall levels of spontaneous RGC activity.

### Ferret VAChT-Sap treatment results in progressive loss of starburst amacrine cells concomitant with defects in correlated retinal activity measured in multi-electrode array recordings

**Figure 4:**
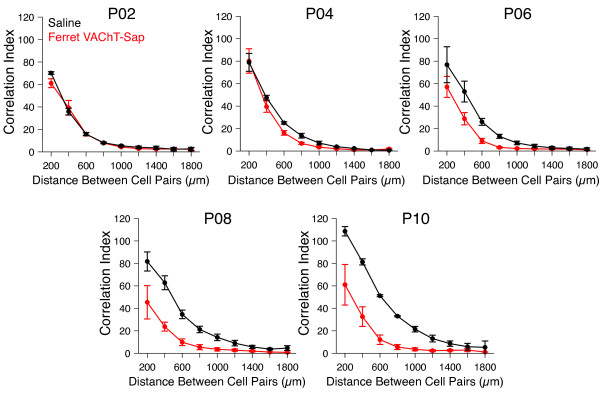
**Ferret VAChT-Sap treatment disrupts spatiotemporal correlations in retinal waves.** Retinal ganglion cell (RGC) pairs in saline control recordings exhibit correlated spontaneous activity that decreases as a function of inter-pair distance (black data points). At P2, treatment with Ferret VAChT-Sap does not perturb the structure of correlations within waves (red data points). Beginning at P4, the structure of correlations between RGC pairs in Ferret VAChT-Sap-treated retinae begins to shift such that by P10, correlations at all inter-pair intervals are reduced in Ferret VAChT-Sap-treated retinae compared to controls. Data points reflect mean values ± SEM; the number of retinae/animals recorded for Ferret VAChT-Sap and Saline conditions at P2 was N = 6 and 5, P4 N = 7 and 4, P6 N = 7 and 6, P8 N = 7 and 6, and P10 N = 4 and 2 respectively.

**Figure 5:**
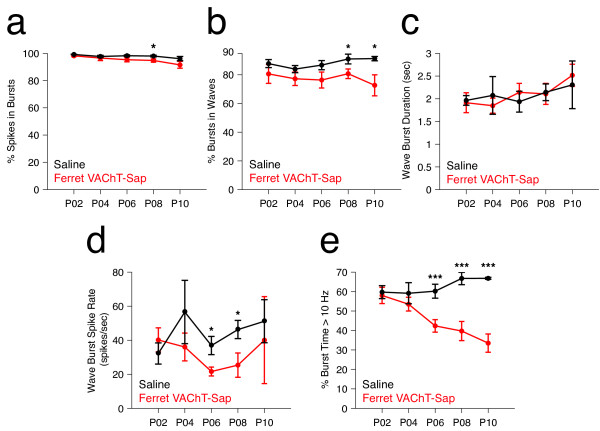
**Effects of Ferret VAChT-Sap treatment on retinal ganglion cell burst properties.** SAC ablation leads to small declines in the fraction of total spikes occurring in bursts **(a)**, as well as the fraction of bursts occurring in waves **(b)**. SAC ablation does not impact the mean duration of bursts in waves **(c)** and the mean spike rate within wave bursts is similar to controls **(d)**. SAC ablation significantly reduces high frequency firing (>10 Hz) within bursts relative to controls **(e)**. Data points reflect mean values ± SEM; the number of retinae/animals recorded for Ferret VAChT-Sap and Saline conditions at P2 was N = 6 and 5, P4 N = 7 and 4, P6 N = 7 and 6, P8 N = 7 and 6, and P10 N = 4 and 2 respectively. Statistics reflect two-tailed *P* values calculated from independent two-sample Student’s t-tests (*P <0.05, ***P <0.001).

Following each physiological recording, recorded retinal tissue was collected and immunolabeled to visualize the number of choline acetyltransferase (ChAT) (+) SACs in each sample. At P2, the number of SACs in Ferret VAChT-Sap-treated retinae (1340.42 ± 170.09 cells/mm^2^; mean ± SEM) was not significantly reduced compared to saline controls (1462.38 ± 199.77 cells/mm^2^; mean ± SEM) consistent with our electrophysiological measurements at this age (Figure 3e). However, at subsequent time points, the number of SACs dropped progressively over development until at P10 SAC density in Ferret VAChT-Sap-treated retinae was 230.40 ± 97.98 cells/mm^2^ (mean ± SEM) compared to 1679.40 ± 350.85 cells/mm^2^ (mean ± SEM) in saline controls, an 86.28% ablation of SACs (Figure 3e). The progressive loss of SACs correlates with the emergence of disrupted patterns of spontaneous retinal activity we recorded at all time points after P2 (the cell counts in Figure 3e directly correspond to recording data presented in Figures 3, 4, and 5).In saline control recordings at all ages recorded, retinal waves produce tightly correlated activity between RGC pairs at short inter-pair distances (<500 μm), with the highest degree of correlation amongst directly neighboring RGCs (Figure 4, black data points). Treatment with Ferret VAChT-Sap did not affect the correlational structure that we observed at postnatal day 2 (P2) relative to saline controls (Figure 4, red data points). However, by P4, correlations between RGC pairs in Ferret VAChT-Sap-treated retinae were lower than those of saline controls for equivalent inter-pair intervals, and this disruption became more pronounced at each subsequent time point investigated (Figure 4). These results are consistent with our dual whole-cell patch recording data showing that Ferret VAChT-Sap reduces correlated RGC firing during this epoch.

### Burst activity in Ferret VAChT-Sap-treated retinae is confined to waves and the majority of spikes are confined to bursts

Consistent with previous studies of normal spontaneous retinal activity, approximately 98% of RGCs in saline control retinae exhibited burst activity during our recordings, and more than 95% of all recorded spikes occurred within RGC bursts (Figure 5a, black data points; Table 1). In retinae treated with Ferret VAChT-Sap, approximately 98% of RGCs exhibited bursts, and the fraction of total spikes occurring in bursts was more than 90% at all ages (Figure 5a, red data points; Table 1). In retinae treated with Ferret VAChT-Sap, although most RGCs exhibited bursts and most spikes were confined to bursts, the percentage of bursts occurring in retinal waves was reduced compared to saline controls (Figure 5b; Table 1). However, for all ages investigated, more than 70% of all bursts were confined to waves following SAC ablation (Table 1). The mean burst frequency increased modestly in Ferret VAChT-Sap-treated retinae compared to saline controls (Table 1) in parallel with the increase in wave frequency observed following SAC ablation. Concomitantly, the mean inter-spike interval within all recorded bursts was higher in Ferret VAChT-Sap-treated retinae than in controls at all time points (Table 1). Thus, although waves and bursts were more frequent following SAC ablation, individual bursts contained fewer spikes and the total level of RGC spike output from immunotoxin-treated retinae was not different from saline controls. Burst duration was unaffected at all ages in Ferret VAChT-Sap-treated retinae, relative to saline controls (Figure 5c; Table 1), and the mean and maximum spike rates for individual bursts were similar over development in both groups (Figure 5d; Table 1).

The pattern of spike activity within RGC bursts may be important for driving strong postsynaptic responses in the dLGN and previous work has implicated high-frequency firing in the refinement of eye-specific projections during development [26, 43–45]. Treatment with Ferret VAChT-Sap significantly decreased the percentage of total burst time that recorded RGCs spent firing at rates greater than 10 Hz (Figure 5e). This decrease in high frequency firing is consistent with the increased burst inter-spike interval (ISI) times measured in Ferret VAChT-Sap-treated retinae compared to controls (Table 1). The features of burst activity described above and presented in Table 1 were similar for bursts occurring in waves and bursts occurring outside of waves.

## Discussion

Our results demonstrate that intraocular ablation of SACs in ferret retinae disrupts patterned spontaneous retinal activity and leads to less correlated spiking between neighboring RGCs, smaller retinal waves, and abnormal burst properties relative to controls. This manipulation preserves overall retinal activity levels and has no impact on eye-specific retinogeniculate segregation. These data suggest that normal spatiotemporal patterns of spontaneous retinal activity present in retinal waves are not critical for retinogeniculate development, and that wave size in particular may not be instructive for eye-specific segregation in the dLGN.

It has been hypothesized that the normal level of RGC activity following SAC ablation is permissive for retinogeniculate patterning guided by molecular cues expressed in RGC axons and postsynaptic targets [14]. However, studies using pharmacological or genetic manipulations have reported the emergence of normal retinofugal projections, despite increases or decreases in overall spontaneous retinal activity levels [38, 43, 46, 47]. Additionally, blocking (or at least reducing) binocular spontaneous retinal activity with intraocular injections of tetrodotoxin delayed, but did not prevent, eye-specific segregation in the ferret [36]. Further, it was recently reported that eye-specific segregation is defective in a mouse mutant exhibiting normal levels of overall retinal activity [39].

Taken together, these data suggest that absolute levels of retinal activity are not predictive of eye-specific retinogeniculate refinement phenotypes. However, our results do support the conclusion that the relative activity of the two eyes influences activity-dependent competition for target territory in the dLGN [12, 38, 48]. Asymmetric interocular ablation of SACs biased competition such that the eye with fewer SACs ceded target territory to the eye with more SACs, even in cases where both eyes had high levels of SAC loss. Interestingly, since SAC ablation did not impact overall levels of retinal spike output, binocular competition appears to have been mediated by disrupted patterns, rather than absolute levels, of spontaneous retinal activity.

The spatiotemporal patterns of correlated spontaneous activity between neighboring RGCs during retinal waves may contribute to Hebbian mechanisms of plasticity during eye-specific circuit refinement [11, 37, 49, 50]. Specifically, the size of retinal waves has been suggested to be the critical feature of patterned spontaneous retinal activity necessary for eye-specific refinement in the thalamus and colliculus [39]. Our results show that SAC ablation reduced retinal wave size and disrupted correlations between RGC pairs at all inter-pair intervals, while eye-specific segregation proceeded normally. These data contradict the hypothesis that retinal wave size is critical for eye-specific retinogeniculate development, an interpretation that was based on MEA recordings from ß2(TG) mice, in which the ß2 subunit of the nicotinic acetylcholine receptor (ß2-nAChR) is restored in the retinae of ß2(KO) mice lacking this subunit entirely [39].

ß2(TG) mice have smaller retinal waves than wild-type mice [39, 51], similar to waves recorded in Ferret VAChT-Sap-treated retinae compared with controls. However, ß2(TG) mice display defects in eye-specific segregation [39] that were never observed in our experiments following SAC ablation. There are several possible explanations for this discrepancy including differences in species (mouse versus ferret), *in vitro* recording conditions (for example, Ames’ medium versus Eagle’s minimum essential medium (MEME)), and the mechanism of the manipulation in the two studies (transgenic gain-of-function rescue in ß2(TG) mice versus pharmacological loss-of-function knockdown of SACs by Ferret VAChT-Sap). More interestingly, in an effort to determine the feature(s) of spontaneous retinal activity necessary for eye-specific segregation, we examined the properties of spontaneous retinal activity recorded in the two studies for comparison with each other and with data reported previously from ß2(KO) mouse retinae (Table 2).

**Table 2:** **Comparison of RGC burst and wave properties recorded at P6 following SAC ablation in ferret retinae with previously published data from ß2KO and ß2(TG) transgenic mice**

	Ferret VAChT-Sap MEME at 37°		Stafford ***et al.*** [ [26] ] : Ames’ at 37°		Xu ***et al.*** [ [39] ] : Ames’ at 37°	
All Bursts	P6 Saline	P6 Ferret VAChT-Sap	Wild-type	ß2 KO	Wild-type	ß2 (TG)
Total Neurons	463	355	258 ± 99	340 ± 161	--	--
Total Neurons Analyzed for Bursts	457	336	--	--	--	--
Bursts Per Neuron	10.37 ± 2.16	12.36 ± 1.22	--	--	--	--
Burst Frequency (bursts/min)	0.69 ± 0.14	0.82 ± 0.08	1.00 ± 0.10	2.10 ± 0.70***	0.63 ± 0.06	0.72 ± 0.08
% Spikes in Bursts	98.18 ± 0.61	95.28 ± 1.60	96.60 ± 1.0	79.60 ± 5.10***	73.52 ± 4.36	68.57 ± 4.01
Maximum Burst Spike Rate	528.04 ± 85.41	528.48 ± 161.02	--	--	--	--
% of Total Burst Time >10 Hz	61.42 ± 3.60	42.82 ± 3.54***	23.20 ± 6.50	11.50 ± 1.90***	--	--
% Bursts in Waves	86.74 ± 3.20	76.34 ± 5.60	88.70 ± 3.60	36.10 ± 7.40***	94.53 ± 2.40	86.99 ± 2.70
Mean Burst Spike Rate (spikes/s)	41.08 ± 7.12	27.84 ± 10.20	--	--	6.18 ± 1.08	5.26 ± 0.87
Burst Interspike Interval (ISI) (ms)	72.20 ± 11.62	132.11 ± 23.90*	192.70 ± 28.70	326.2 ± 59.9***	360 ± 120	650 ± 180*
Mean Spike Rate (spikes/s)	0.91 ± 0.31	0.69 ± 0.14	0.40 ± 0.09	0.43 ± 0.14	0.18 ± 0.04	0.18 ± 0.03
Burst Duration (s)	1.94 ± 0.23	2.25 ± 0.19	--	--	2.47 ± 0.47	3.76 ± 1.60
	**P6 Saline**	**P6 Ferret VAChT-Sap**	**Wild-type**	**ß2 KO**	**Wild-type**	**ß2 (TG)**
Wave Bursts						
Burst Duration	1.93 ± 0.23	2.14 ± 0.20	2.96 ± 0.25	3.18 ± 0.57	--	--
Burst Spike Rate	37.14 ± 5.34	21.63 ± 2.60*	--	--	--	--
Burst (ISI)	67.71 ± 9.09	131.06 ± 21.76**	189.20 ± 27.40	302.90 ± 55.50***	--	--
Burst Spike Rate from ISI	15.98 ± 2.24	8.11 ± 1.33**	--	--	--	--
Interburst Interval (IBI)	154.43 ± 27.59	143.91 ± 10.34	--	--	--	--
Maximum Burst Spike Rate	529.49 ± 66.71	456.35 ± 140.18	--	--	--	--
% Neurons Bursting Per Second In Waves	5.01 ± 0.35	3.04 ± 0.15***	--	--	--	--
Bursts Per Neuron Per Wave	1.12 ± 0.07	1.17 ± 0.14	1.02 ± 0.02	1.18 ± 0.12**	--	--
Percent Burst Time >10 Hz	60.17 ± 3.60	42.37 ± 3.22***	78.30 ± 4.20	41.60 ± 7.20***	--	--
Non-Wave Bursts						
Burst Duration	1.99 ± 0.24	2.34 ± 0.20	2.68 ± 0.18	2.38 ± 0.40*	--	--
Burst Spike Rate	46.14 ± 10.29	31.83 ± 17.60	--	--	--	--
Burst ISI	77.88 ± 14.38	144.92 ± 32.94*	204.40 ± 33.10	338.10 ± 66.10***	--	--
Burst Spike Rate from ISI	46.14 ± 29	31.83 ± 17.60	--	--	--	--
Maximum Burst Spike Rate	496.95 ± 117.20	583.86 ± 188.27	--	--	--	--
Percent Burst Time >10 Hz	59.43 ± 4.75	40.89 ± 3.93**	21.70 ± 4.20	58.10 ± 7.20***	--	--
	**P6 Saline**	**P6 Ferret VAChT-Sap**	**Wild-type**	**ß2 KO**	**Wild-type**	**ß2 (TG)**
Wave size (% of channels)	33.48 ± 2.03	13.33 ± 0.50***	--	--	41.32 ± 8.32	9.50 ± 1.37***
Wave frequency (per min)	2.26 ± 0.56	4.43 ± 0.90	1.66 ± 0.30	0.80 ± 0.28***	0.82 ± 0.09	0.85 ± 0.16
Wave duration (s)	--	--	13.00 ± 2.80	10.80 ± 3.30	10.05 ± 1.42	5.42 ± 0.63***

The table list mean values ± SEM for all parameters recorded. Statistics reflect two-tailed *P* values calculated from independent two-sample Student’s t-tests (*P <0.05, **P <0.01, ***P <0.001). P6, postnatal day 6; P10, postnatal day 10; ISI, interspike interval; IBI, interburst interval.

During normal retinal waves a refractive period of quiescence following RGC bursts in each wave reduces the likelihood of coincident signaling between the two eyes, and may facilitate independent recognition of eye-specific inputs by postsynaptic targets. In ß2(KO) retinae a high fraction of the total spike activity of RGCs occurs as isolated spiking events outside of bursts and waves. This increases the probability that postsynaptic cells in the binocular zone of overlap receive coactive inputs from both eyes and may thereby prevent refinement of overlapping projections in the dLGN [26, 39]. Following SAC ablation in the ferret retina, burst activity was primarily confined to waves, and more than 95% of total spikes were confined to bursts. In ß2(TG), however, the percentage of total spikes occurring in bursts is low, and is comparable to that seen in ß2(KO) mice (Table 2) consistent with a role for uncorrelated spike activity in the development or maintenance of incorrectly targeted eye-specific projections in the dLGN. Interestingly, treating ß2(TG) mice with intraocular injections of cAMP analogues, shown previously to increase the size and frequency of retinal waves in ferret retinae [38, 52], rescues eye-specific segregation defects consistent with the hypothesis that normal wave size is important for retinogeniculate development [39]. However, physiological recording data from ß2(TG) retinae in the presence of cAMP analogues were not presented in that study, and it remains unclear which feature(s) of spontaneous activity were restored by this manipulation to enable eye-specific segregation. Experiments recording spontaneous retinal activity patterns in ß2(TG) mouse retinae in the presence of cAMP analogues are necessary to address this issue.

An alternative interpretation of why eye-specific segregation is normal following SAC ablation in ferret retinae, but not in ß2(TG) mice, relates to the time course of the manipulations in the two studies. SAC ablation causes delayed disruption of spontaneous retinal activity patterns beginning between P2 and P4 in the ferret [14]. It is possible that an early period of normal wave activity is sufficient to mediate initial activity-dependent remodeling events, or responses to molecular cues in the dLGN that drive eye-specific segregation, despite later abnormalities in spontaneous retinal activity. In ß2(TG) mice, spontaneous retinal activity patterns may be disrupted from birth and thereby lead to defects in eye-specific segregation. However, a complete picture of the time course of activity in these animals has not been presented [39]. In light of the developmentally-regulated expression of guidance factors on RGC axons and within the dLGN [15, 16, 29, 53], it is important that future studies document retinal activity patterns over the time course of retinogeniculate development.

Lastly, it is equally important to consider the possible mechanisms of retinal wave generation following SAC ablation in ferrets compared with ß2-nAChR rescue in ß2(TG) mice. Spontaneous retinal activity in ß2(TG) mice is sensitive to a nicotinic antagonist and it is assumed that waves in these mice are cholinergic and driven by SACs [39]. Although ß2-nAChR transcripts are expressed throughout the ganglion cell layer of ß2(TG) animals where the cell bodies of On SACs also reside, the small size of retinal waves in these animals was attributed to the selective rescue of the ß2-nAChR subunit only in RGCs and not in SACs [39]. This expression pattern would allow RGCs to receive cholinergic signals from SACs while maintaining the functional isolation of individual SACs from one another, thereby preventing the normal propagation of retinal waves within a larger coupled SAC network. Similarly, SAC ablation in our experiments decouples the cholinergic network and tends to isolate the surviving SACs from nearby neighbors. Importantly, wave size in retinae treated with Ferret VAChT-Sap is similar to wave size observed in ß2(TG) mice. Individual SACs are capable of initiating retinal waves [41] and it is possible that, in both manipulations, cholinergic signaling from decoupled SACs underlies the smaller wave events. Moreover, we did not observe a compensatory increase in retinal wave size or velocity as shown previously in retinae that revert to gap junction-mediated retinal wave generation when cholinergic networks are perturbed [25, 26, 42]. Thus, while our data do not rule out compensatory mechanisms of retinal wave generation in Ferret VAChT-Sap-treated retinae, they are consistent with the hypothesis that the remaining SACs spared from immunotoxin ablation give rise to the wave activity observed in our experiments.

To evaluate different features of spontaneous retinal activity for correlation with eye-specific segregation defects, or the lack thereof, we compared our current results to published data from other manipulations of spontaneous retinal activity (Table 2). Manipulations of SAC signaling that disrupt eye-specific retinogeniculate refinement impact RGC bursting [25, 26, 43, 54], consistent with some feature(s) of burst activity being important for normal refinement mechanisms. RGCs of retinae treated with the cholinergic agonist epibatidine (EPI) [54] and retinae from ß2(KO) mice [25, 26] both show increased RGC burst frequency, raising the probability of coincident firing between the two eyes [55]. In our current experiments, treatment with Ferret VAChT-Sap increased burst frequency with no impact on normal eye-specific segregation. Further, binocular treatment with cAMP analogues increases wave and burst frequency without disrupting eye-specific development [38]. Conversely, burst frequency is normal in ß2(TG) mouse retinae, while eye-specific segregation is disrupted [39]. Thus, changes in burst frequency do not appear to correlate with eye-specific segregation phenotypes.

The number of spikes per RGC burst may be important for Hebbian refinement of eye-specific circuits [26, 55], with stronger bursts evoking postsynaptic responses and calcium influx that trigger presynaptic and postsynaptic refinement. ß2(KO) and ß2(TG) mice each exhibit fewer spikes per burst than wild-type mice, consistent with stronger bursts being important in the refinement process [25, 26]. However, Ferret VAChT-Sap-treated retinae have fewer spikes per burst in individual RGCs with no effects on eye-specific refinement. Further, EPI treatment, which prevents eye-specific segregation, leads to an increase in spikes per burst in many RGCs [54]. Comparison across these studies suggests there is no correlation between the number of spikes per burst and eye-specific retinogeniculate refinement phenotypes.

The temporal frequency of spikes within bursts contributes to postsynaptic depolarization [56], and high frequency firing may drive calcium influxes necessary for initiating retinogeniculate synaptic potentiation or depression [2, 57, 58]. ß2(KO) mice have reduced RGC spiking at rates greater than 10 Hz [25, 26, 43], and retinae treated with EPI show similar defects [54], consistent with a role for high-frequency firing in eye-specific retinogeniculate refinement [43, 45]. In contrast, we recorded a significant decrease in the percentage of total burst time RGCs spent firing over 10 Hz following ablation of SACs, with no impact on eye-specific refinement during development. This effect is comparable to that seen in ß2(KO) retinae [26], demonstrating that high-frequency firing in bursts is not required for eye-specific segregation and likely cannot account for the retinogeniculate defects seen in ß2(KO) mice. Additionally, connexin 36/45 double-knockout mutant mice exhibit significant increases in the percentage of time RGCs fire at rates greater than 10 Hz, concomitant with defects in eye-specific segregation [45]. Thus, there appears to be no correlation between the fraction of time RGCs spend firing at high frequencies and defects in eye-specific segregation. However, the same caveats discussed previously for the comparison to ß2(TG) mice apply in these comparisons to ß2(KO) and connexin 36/45 dKO mice. The decrease in high-frequency firing in Ferret VAChT-Sap-treated retinae is not apparent at early ages (P2 to P4), and a period of normal high-frequency activity may be sufficient to drive early remodeling events in the developing retinogeniculate pathway.

There is an important final qualification regarding the conclusions we have drawn from comparing across studies in the literature: our analysis assumes the veracity of published recording data from laboratories using different animal models, recording conditions, and ages. The response properties of isolated retinae *in vitro* depend upon each of these factors, as well as on recording temperature and ambient luminance, among others. Accordingly, conclusions based upon cross-study comparisons should be interpreted as suggestive hypotheses for future experiments *in vivo*.

## Conclusions

In summary, we have shown that patterned spontaneous retinal activity can be significantly disrupted without affecting the normal development of eye-specific retinogeniculate projections. A comparison of our current results to previously published data from genetic and pharmacological manipulations of spontaneous retinal activity suggests that wave size, absolute activity levels, normal correlations between RGC pairs, RGC burst frequency, high frequency RGC firing during bursts, and the number of spikes per RGC burst are features of spontaneous retinal activity that do not strictly correlate with eye-specific refinement phenotypes seen across these studies. By contrast, abnormal eye-specific retinogeniculate segregation phenotypes are found whenever there is a high fraction of total RGC spiking occurring outside of burst activity. The axons of RGCs with isolated, asynchronous spike activity outside of bursts may fail to participate in normal mechanisms of synapse formation/elimination dependent on strong synaptic drive, presynaptic/postsynaptic calcium influx elicited during burst activity, and/or molecular regulation of synaptic development and plasticity [59, 60]. In the future, the ability to precisely manipulate patterns of retinal activity during development [61], combined with sophisticated methods of recording activity patterns *in vivo*[62], will improve our understanding of how spontaneous retinal activity contributes to the development of connections from the eyes to the brain.

## Methods

### Animals

Timed-pregnant jill ferrets were obtained from Marshall Farms (New Rose, New York, United States). Postnatal day zero (P0) was defined as the day of birth. Pups of both sexes were used in the experiments. All experimental procedures were performed in accordance with the National Institutes of Health guidelines under protocols approved by the Institutional Animal Care and Use Committee (IACUC) of the University of California, Davis (#16676 and #18342).

### Immunotoxin generation

A previous study from our laboratory used a VAChT-Saporin immunotoxin to ablate SACs of the ferret retina early in postnatal development [14]. This immunotoxin recognized an antigenic domain on the C-terminus of the mouse vesicular acetylcholine transport protein (VAChT), which is the same as that employed in commercial anti-VAChT antibody design. Although the previous immunotoxin used by Huberman *et al.*[14] successfully ablated SACs of the ferret retina, the antibody did not yield positive staining for SAC dendrites in ferret retinal tissue (Dr A Huberman, personal communication). Further, when we cloned the Ferret VAChT gene we discovered only 50% similarity (10 out of 20 amino acids) between mouse and ferret in the commercial antigenic domain against which the previous immunotoxin was made (data not shown). When we tested a custom antibody designed against this ferret-specific epitope we found no specific staining co-localizing with the ChAT (+) sublaminae in the ferret retina (data not shown). Therefore, we opted to generate a novel antibody specific for ferret VAChT.

The coding region for the C-terminal domain of the VAChT protein was cloned from a ferret cDNA library using standard molecular techniques. The primers used were: Sense primer 5′-TACGCGCTCGGGCCCATAGT-3′ and Antisense primer 5′-TGGAGGAGAAGCGGGTCT GCT-3′. PCR products were sequenced and suitable antigenic regions were determined. Rabbits were immunized with a synthetic 20 AA peptide (sequence: RRSRSERDVLLDEPPQGLYD) corresponding to an epitope in the C-terminal coding region of our cloned *VAChT* sequence (Open Biosystems, Huntsville, Alabama, United States). Whole-animal serum was affinity-purified and shipped to our laboratory for verification of antibody specificity (see Immunohistochemistry in this section). SAC-specific affinity-purified ferret VAChT antibody was conjugated to Saporin toxin (Advanced Targeting Systems, San Diego, California, United States).

### Immunotoxin treatment

The intravitreal injection protocol was adapted from Speer *et al.*[4]. P0 ferrets were anaesthetized with inhalant isoflurane (Baxter Healthcare, Deerfield, Illinois, United States) mixed with oxygen and 1 to 2 μl of Ferret VAChT-Sap (0.88 μg/μl) was injected into the eye. This protocol was repeated on P1. Control eyes were injected with equivalent volumes of 0.9% saline (Baxter Healthcare, Deerfield, Illinois, United States). Animals for MEA and dual-patch recording were injected with Ferret VAChT-Sap in the left eye and saline in the right eye, thereby providing within-animal controls. Animals for anatomical study of retinogeniculate development were injected binocularly with either Ferret VAChT-Sap or saline of equivalent volumes in the two eyes.

### Patch clamp recordings of neighboring retinal ganglion cell pairs

Paired recordings were obtained as described previously [63]. Briefly, retinae were removed and stored at room temperature in MEME (M7278 Sigma-Aldrich, St. Louis, Missouri, United States) bubbled with 95% O_2_ and 5% CO_2_. A small piece of the retina was placed ganglion cell layer up over a 2 mm hole in a piece of filter paper and secured in the recording chamber. Cells were visualized through a 40× objective mounted on a fixed-stage upright epifluorescence microscope (Nikon, Tokyo, Japan). The tissue was continuously perfused with bubbled MEME which was gravity-fed into the recording chamber and maintained at 35°C. Current-clamp recordings were made with a 200B Axopatch amplifier (Molecular Devices, Sunnyvale, California, United States). The data were low pass filtered at 1 kHz and digitized at 4 kHz before digital storage for off-line analysis. For dual patch recordings, after attaining whole cell configuration in both cells, the resting membrane potentials of each cell were monitored throughout the recording. Spontaneous activity was typically recorded for 5 to 20 minutes. The patch electrode contained 0.5% Lucifer Yellow or Alexa Fluor (488 or 568, 10 mM) (Life Technologies, Grand Island, New York, United States) which allowed fluorescent visualization of neurons following recording. Each cell included in the data analysis was morphologically confirmed to be an RGC by the presence of an axon in the nerve fiber layer. Recordings were made from five RGC pairs in saline controls and 10 RGC pairs from Ferret VAChT-Sap-treated retinae. Somas of RGC pairs were typically 15 to 35 μm apart. In some cases, only one cell in a paired recording was successfully patched, and these data were included in our examination of mean firing frequency (16 total cells for saline controls and 32 cells for Ferret VAChT-Sap treatment). The number of retinae/animals recorded for Ferret VAChT-Sap and Saline conditions at P2 was N = 2 and 3, P6 N = 4 and 3, P9 N = 1 and 1, and P10 N = 1 and 1 respectively.

### Cross-correlation analysis and mean firing frequency

The degree of synchrony between members of a pair of simultaneously recorded cells was characterized as described previously [63]. The correlation coefficient between the instantaneous activities of the two cells at different temporal offsets was calculated. Spikes and depolarizations were extracted by computing the derivative, setting a threshold, and then smoothing the resulting activity train with a 500-msec-wide Gaussian window (σ = 250 msec). The correlation coefficient at offset τ was calculated as
1

where *ρ*_1_ and *ρ*_2_ denote the instantaneous activities of cells one and two, respectively, *COV*(*ρ*_1_, *ρ*_2_, *τ*) is the covariance between *ρ*_1_ and *ρ*_2_ with a temporal offset of *τ*, and *VAR*(*ρ*_1_) is the variance in activity. Thus, the correlation coefficient is the normalized covariance, yielding a number from -1 to +1, where +1 corresponds to the maximum synchrony and -1 to maximal asynchrony. Mean firing frequencies were also calculated for all cells. In addition to the analysis of the data collected in the current study, equivalent quantification was carried out for the raw data acquired in a previous study using a different immunotoxin (Huberman *et al.*[14]), allowing us to directly compare the data from the two studies.

### Multi-electrode array recordings

The procedures for retinal dissection for MEA recording have been described previously [54]. Neonatal ferrets were administered a lethal dose of Euthana-6 (0.1 ml, pentobarbital sodium, Western Medical Supply, Arcadia, California, United States). Animals were enucleated and the retinae were removed and maintained in buffered, oxygenated MEME (M7278, Sigma-Aldrich, St. Louis, Missouri, United States) at room temperature. Sections of retinae measuring approximately 5 to 8 mm^2^ were cut for recording on the MEA. Retinal sections were placed ganglion cell layer down onto a 60-channel MEA (Multi-Channel Systems, Tubingen, Germany), held fixed by a dialysis membrane (Spectrapore 132130, Spectrum, Los Angeles, California, United States), and superfused with buffered/oxygenated MEME at 1 to 2 ml/min at 37°C. Array electrodes were 30 μm in diameter, arranged on an 8 × 8 rectilinear grid with 200-μm inter-electrode spacing. At this inter-electrode spacing, the signal of a given cell appeared on only one electrode, so each cell was assigned the coordinates of the electrode that recorded its signal. Analog data were acquired at 20 kHz per channel simultaneously from each of the 60 electrodes. Following experimental setup, retinae were allowed to acclimate for 5 to 20 minutes on the MEA. On emergence of retinal wave epochs, recordings were performed for a period of 15 to 20 minutes, during which time overall firing rates of the RGC ensemble appeared stable. Ferret VAChT-Sap-treated retinae were recorded first after which the MEA was cleaned. Control saline-treated retinae were then prepared. The number of retinae/animals recorded for Ferret VAChT-Sap and Saline conditions at P2 was N = 6 and 5, P4 N = 7 and 4, P6 N = 7 and 6, P8 N = 7 and 6, and P10 N = 4 and 2.

### Spike identification

Spike sorting and cell identification were performed identically to that in Sun *et al.*[54]. Before sorting spike events, the data were digitally filtered with a 125-Hz high-pass filter (four-pole Butterworth) (Multi-channel Systems, Tubingen, Germany). A threshold of 6 SD was set for each channel and 1 ms of data before a threshold-crossing event, and 4 ms after the threshold event were stored for each negative-slope event. These candidate spike waveforms were then sorted with the OfflineSorter (Plexon, Denton, Texas, United States) using the first three principal components of the spike waveforms. Coincident events within 0.5 ms of one another that occurred on all electrodes were attributed to perfusion noise and removed. Clusters were first identified using an expectation-maximization cluster algorithm [64] and then manually edited for clustering errors. Typically, each electrode recorded the activity of one to three cells.

### Retinal ganglion cell burst correlation analysis

Burst analysis, including automated wave versus non-wave burst detection, and analysis of correlated spike activity were performed as in Stafford *et al.*[26]. Two-sample independent Student’s t-tests were applied to the data for group comparison.

### Wave frequency and size analysis

Waves were visualized from the raw multi-electrode analog data and defined as coincident bursting propagating on four or more neighboring electrode sites. Individual waves were counted (frequency) and the number of channels activated during each wave was recorded (size). For analysis of wave bursts versus non-wave bursts, waves were automatically classified and quantified as in Stafford *et al.*[26].

### Retinal immunohistochemistry

Following MEA recordings, dissected retinae and recorded retinal pieces were collected and fixed briefly (10 minutes) in 4% paraformaldehyde (PFA) (Sigma-Aldrich, St. Louis, Missouri, United States) in 0.1 M phosphate buffer. Following fixation, retinae were washed several times in 0.1 M phosphate buffer and processed for whole-mount immunohistochemistry. Relieving cuts were made to allow the retina to lay flat. Retinae were incubated in blocking solution (10% donkey serum, 0.3% Triton-X 100 in 0.1 M phosphate buffer (Jackson Immunoresearch, West Grove, Pennsylvania, United States) for four hours, then transferred to primary antibodies (1:50 goat anti-ChAT; Millipore, Billerica, Massachusetts, United States; 1:100 mouse NeuN; Millipore, Billerica, Massachusetts, United States) for 48 hours at 4°C, washed in 0.1 M phosphate buffer overnight, transferred to secondary antibody (1:200 donkey anti-goat Alexa 488, 1:200 donkey anti-mouse Alexa 594; Life Technologies, Grand Island, New York, United States) for 2 hours at room temperature, washed in 0.1 M phosphate buffer five times for 30 minutes each time, mounted, and cover-slipped with Vectashield (Vectorlabs, Burlingame, California, United States).

For thin-section retinal immunohistochemistry, retinae were dissected and fixed in 4% PFA for 10 to 20 minutes, rinsed in 0.1 M PB for 20 minutes, immersed in 30% sucrose, frozen in Optimal Cutting Temperature compound (OCT) (Electron Microscopy Sciences, Hatfield Pennsylvania, United States), cryosectioned at 18 μm, and mounted on positive-coated slides. Sections were incubated in blocking solution (10% donkey serum, 0.3% Triton-X 100 in 0.1 M phosphate buffer) for four hours, transferred to primary antibodies in blocking solution (1:50 goat anti-ChAT; Millipore, Billerica, Massachusetts, United States; 1:100 mouse anti-NeuN; Millipore, Billerica, Massachusetts, United States); 1:1,000 mouse anti-Calbindin; Sigma-Aldrich (St. Louis, Missouri, United States); 1:500 rabbit anti-Calretinin, Millipore, Billerica, Massachusetts, United States; 1:500 rabbit anti-VAChT, custom antibody (see Immunotoxin generation); Brn3a 1:50, Millipore, Billerica, Massachusetts, United States; 1:1,000 rabbit anti-Recoverin; Millipore, Billerica, Massachusetts, United States) overnight at 4°C, washed five times for 30 minutes each and then again overnight in 0.1 M phosphate buffer, transferred to secondary antibodies in blocking solution (1:200 donkey anti-mouse Alexa 594, 1:200 donkey anti-rabbit Alexa 488, 1:200 donkey anti-goat Alexa 488 or 594; Life Technologies, Grand Island, New York, United States) for 2 hours at room temperature, washed in 0.1 M phosphate buffer five times for 30 minutes per time, and cover-slipped with Vectashield plus DAPI (Vectorlabs, Burlingame, California, United States).

Thin-sectioned immunostained retinae were imaged and 1-mm-long images of retinae were cropped at a distance of 500 μm on either side of the optic nerve head. Two cropped fields from each imaged retinal section were acquired and four to six sections per retina were imaged for each retinal label analyzed. Individual cells within each image were counted using the Cell Counter PlugIn in ImageJ [65]. Counts were made from three saline control retinae and four Ferret VAChT-Sap-treated retinae. For whole-mount analysis of ChAT(+) cells, a focal plane midway between the ganglion cell layer and the inner nuclear layer was used so as to include all SACs in the density measurements. Counts were made from seven saline control retinae and 10 Ferret VAChT-Sap-treated retinae (two at 1.76 μg, three at 2.64 μg, and five at 3.52 μg).

### Retinogeniculate labeling and analysis

Anterograde dye-tracing of retinogeniculate afferents and analysis of eye-specific segregation were performed as in Speer *et al.*[4]. Sections were cut at 40 μm in the horizontal plane of section through the center of the dLGN. In each animal, five to seven sections from each side of the dorsal thalamus were thresholded at 30% above background, a value previously determined to result in the most accurate assessment of anterograde tracer signal in the dLGN [4]. Thresholded images were quantified in ImageJ [65]. A total of 14 Ferret VAChT-Sap-treated animals (four symmetric ablation and 10 asymmetric ablation) and two saline-treated animals were included in this analysis.

## Authors’ information

Colenso M Speer and Chao Sun are co-first authors. This article is dedicated in remembrance of Dr. Barbara Chapman who passed away February 11, 2013.

## Electronic supplementary material

Additional file 1: **Ferret-specific vesicular acetylcholine transport protein-saporin (Ferret VAChT-Sap) immunotoxin treatment results in dose-dependent ablation of starburst amacrine cells (SACs).** Whole-mount immunohistochemistry shows choline acetyltransferase (ChAT) (+) SACs (green) and NeuN (+) neurons (magenta) in the ganglion cell layer (GCL) imaged from the same retinal locations at postnatal day 10 (P10) (a). Treatment with increasing doses of Ferret VAChT-Sap leads to dose-dependent loss of SACs without significant loss of NeuN (+) neurons in the GCL (a-c). Quantification shows mean + SEM measured for all four retinal quadrants (dorsal, ventral, nasal, and temporal). Counts were made from seven saline control retinae and ten Ferret VAChT-Sap-treated retinae (two at 1.76 μg, three at 2.64 μg, and five at 3.52 μg). Statistics reflect a one-way ANOVA with Bonferroni *post-hoc* correction for group means averaged across all retinal quadrants (***P <0.001). All group mean comparisons with no corresponding asterisks did not reach significance. Scale bars in (a) are 100 μm. (PDF 6 MB)

Additional file 2: **Ferret VAChT-Sap treatment does not ablate retinal ganglion cells.** Starburst amacrine cells (SACs) and their dendrites (magenta) are immunopositive for choline acetyltransferase (ChAT). Ferret-specific vesicular acetylcholine transport protein antibody (Ferret VAChT) labels SAC dendrites (green) in the inner plexiform layer (IPL) shown here at postnatal day 10 (P10) (a). Treatment with Ferret VAChT-Sap leads to ablation of SACs and a loss of staining for SAC dendrites (a,b). Staining for Brn3a and NeuN (green) labels retinal ganglion cells (RGCs), which are not ablated by Ferret VAChT-Sap treatment (c-f). Scale bars are 100 μm. Quantification shows mean + SEM; counts were made from three saline control retinae and four Ferret VAChT-Sap-treated retinae. Statistics reflect two-tailed *P* values calculated from independent two sample Student’s t-tests. In a-f, ‘Merge’ panels show counterstaining with DAPI (blue) to reveal retinal cytoarchitecture. GCL, ganglion cell layer; INL, inner nuclear layer; IPL, inner plexiform layer; NS, not significant; ONL, outer nuclear layer; OPL, outer plexiform layer; PRL, photoreceptor layer. (PDF 6 MB)

Additional file 3: **Ferret VAChT-Sap treatment ablates horizontal cells and decreases overall retinal thickness.** Calbindin labels horizontal cells (HCs, green) in the inner nuclear layer (INL), as well as their dendrites in the outer plexiform layer (OPL) at postnatal day 10 (P10) (a). HCs are significantly ablated by Ferret VAChT-Sap treatment (a,d). Calretinin also labels HCs as well as a heterogeneous population of neurons in the ganglion cell layer (GCL) (b, green images). Ferret VAChT-Sap treatment leads to loss of calretinin (+) cells in the INL (b,e; calretinin (+) cells in the GCL were not quantified). Recoverin labels photoreceptor somas and outer segments in the outer nuclear layer (ONL) and photoreceptor layer (PRL) respectively, as well as axonal processes of some bipolar cells terminating in the inner plexiform layer (IPL) (c, green images). Ferret VAChT-Sap-treated retinae show qualitatively similar recoverin (+) labeling (c). The absolute thickness of the retinal sheet in Ferret VAChT-Sap-treated retinae is reduced relative to saline controls (f). Scale bars are 100 μm. Quantification shows mean ± SEM; counts were made from three saline control retinae and four Ferret VAChT-Sap-treated retinae. Statistics reflect two-tailed *P* values calculated from independent two sample Student’s t-tests. In a-c, ‘Merge’ panels show counterstaining with DAPI (blue) to reveal retinal cytoarchitecture. (PDF 5 MB)

Additional file 4: **Effects of Ferret VAChT-Sap treatment on retinal cell loss.** Supplemental results word document. (DOCX 30 KB)

## Abbreviations

ß2(KO): A line of transgenic mice with the ß2 subunit of the nicotinic acetylcholine receptor knocked out
ß2(TG): A line of transgenic mice in which ß2 subunit of the nicotinic acetylcholine receptor expression is limited to the ganglion cell layer of the retina
ß2-nAChR: The ß2 subunit of the nicotinic acetylcholine receptor
ChAT: Choline acetyltransferase
dLGN: Dorsal lateral geniculate nucleus
MEME: Eagle’s minimum essential media
EPI: Epibatidine
MEA: Multi-electrode array
NS: not significant
OCT: Optimal Cutting Temperature Medium
P0: Postnatal day zero
P2: Postnatal day two
P4: Postnatal day four
P6: Postnatal day six
P8: Postnatal day eight
P10: Postnatal day 10
PFA: Paraformaldehyde
RGC: Retinal ganglion cell
SAC: Starburst amacrine cell
Sap: Saporin
SEM: standard error of the mean
SRA: Spontaneous retinal activity
VAChT: Vesicular acetylcholine transporter.
