# Supplementary figures and images for: Eye-specific retinogeniculate segregation proceeds normally following disruption of patterned spontaneous retinal activity
Source: Neural Dev. 2014 Nov 7;9:25. doi: 10.1186/1749-8104-9-25 (PMC4289266; doi:10.1186/1749-8104-9-25)

# **a** Dose-dependent Ablation of SACs by Ferret VACHT-Sap

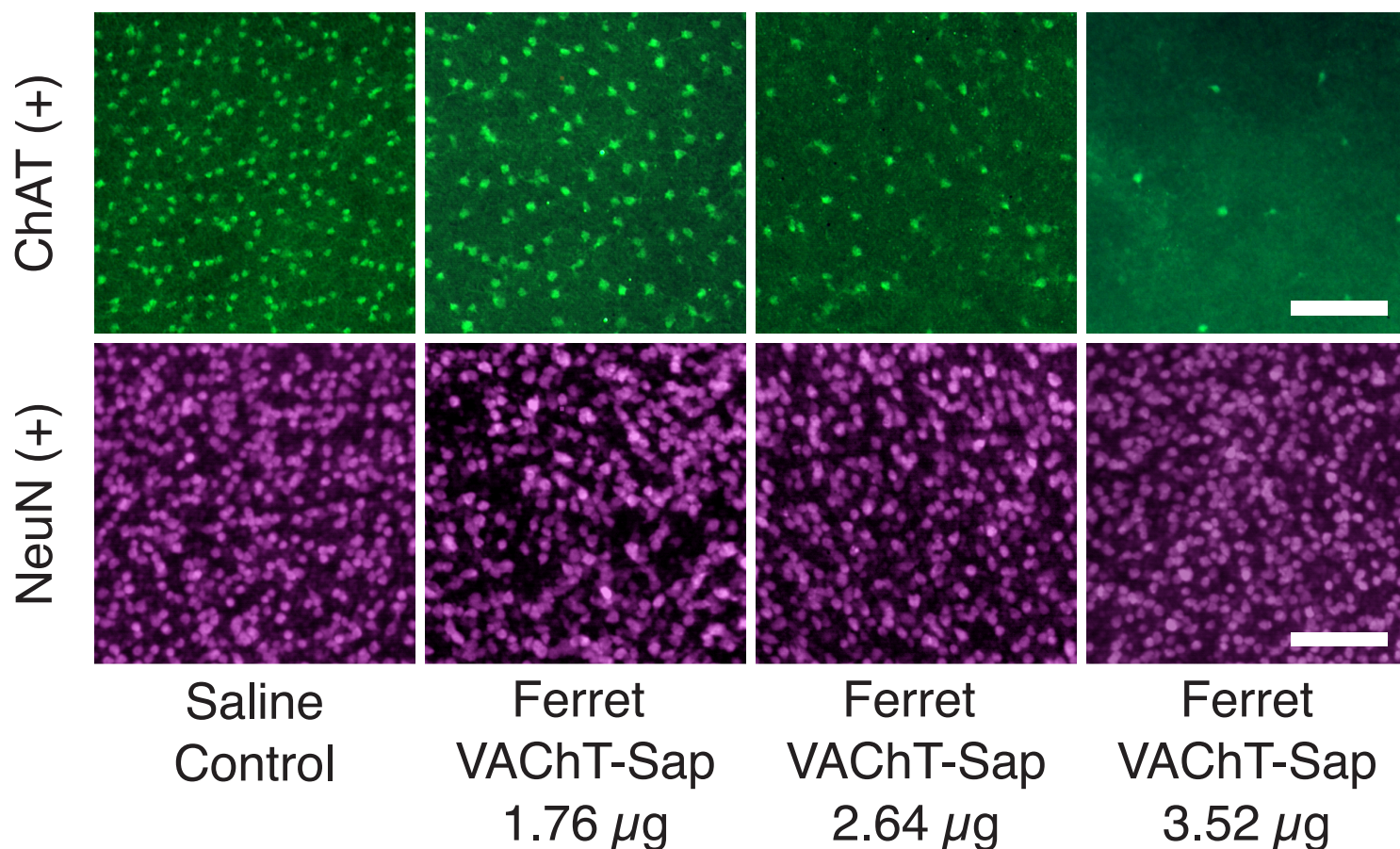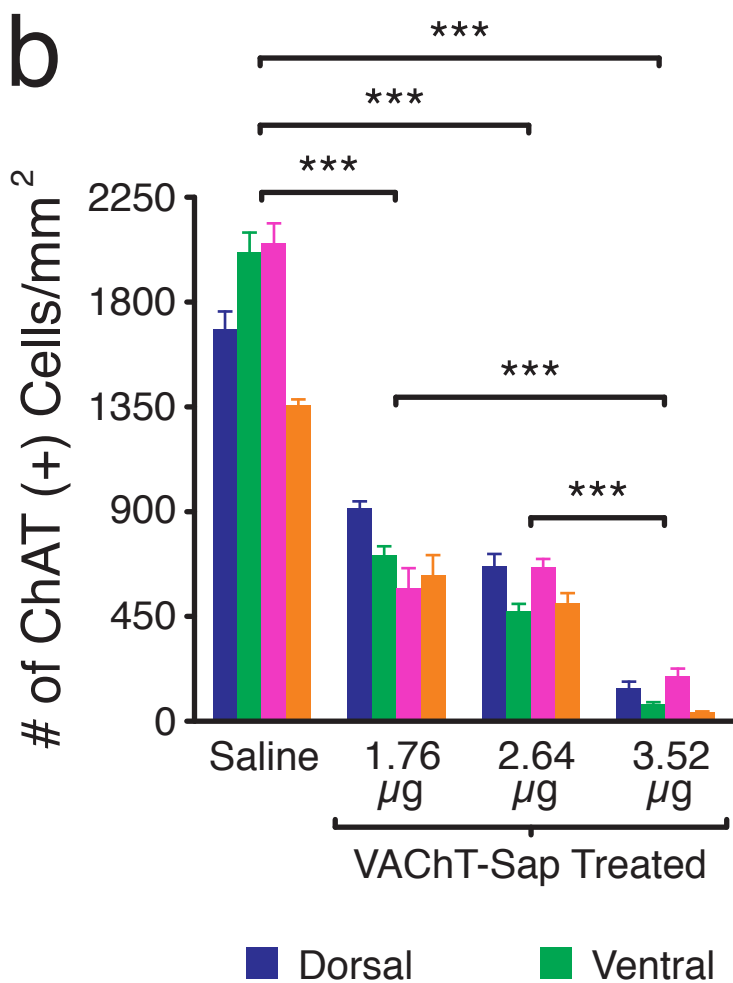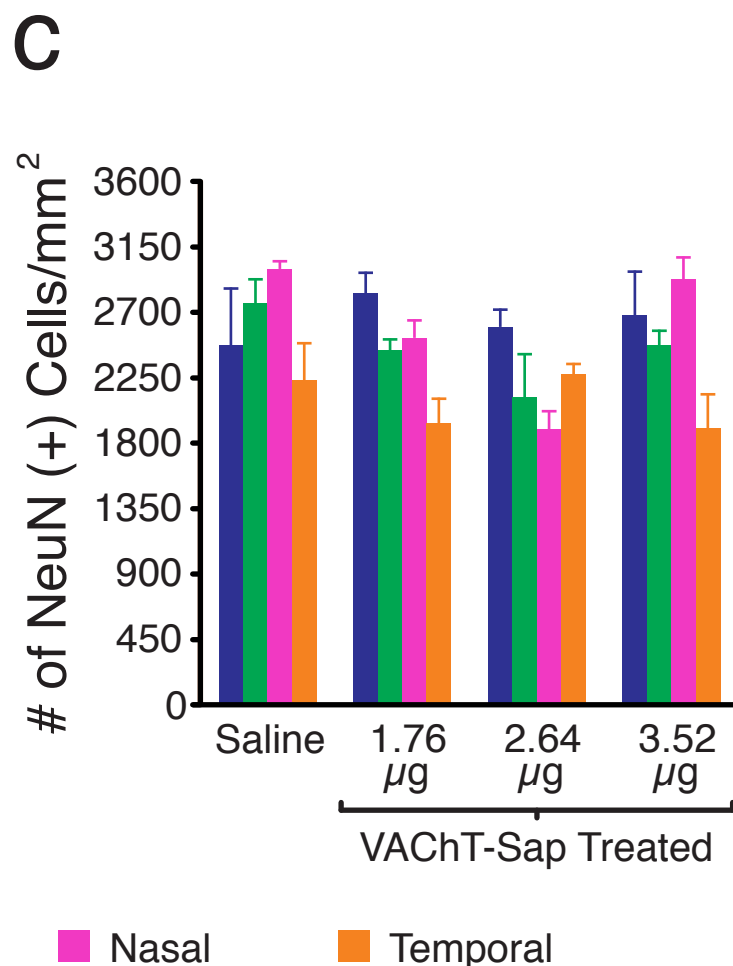

Supplement: Supplementary file 1 — Additional file 1: Ferret-specific vesicular acetylcholine transport protein-saporin (Ferret VAChT-Sap) immunotoxin treatment results in dose-dependent ablation of starburst amacrine cells (SACs). Whole-mount immunohistochemistry shows choline acetyltransferase (ChAT) (+) SACs (green) and NeuN (+) neurons (magenta) in the ganglion cell layer (GCL) imaged from the same retinal locations at postnatal day 10 (P10) (a). Treatment with increasing doses of Ferret VAChT-Sap leads to dose-dependent loss of SACs without significant loss of NeuN (+) neurons in the GCL (a-c). Quantification shows mean + SEM measured for all four retinal quadrants (dorsal, ventral, nasal, and temporal). Counts were made from seven saline control retinae and ten Ferret VAChT-Sap-treated retinae (two at 1.76 μg, three at 2.64 μg, and five at 3.52 μg). Statistics reflect a one-way ANOVA with Bonferroni post-hoc correction for group means averaged across all retinal quadrants (***P <0.001). All group mean comparisons with no corresponding asterisks did not reach significance. Scale bars in (a) are 100 μm. (PDF 6 MB) [file 13064_2014_271_MOESM1_ESM.pdf]

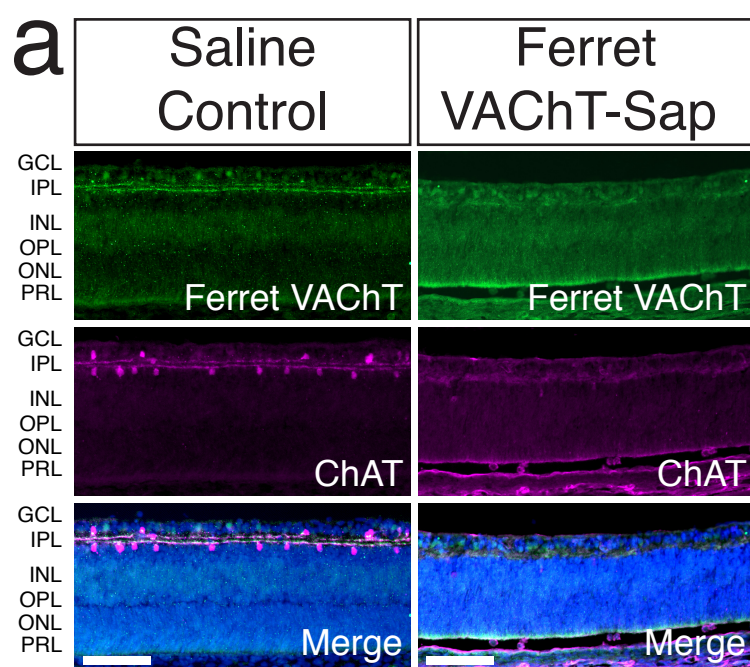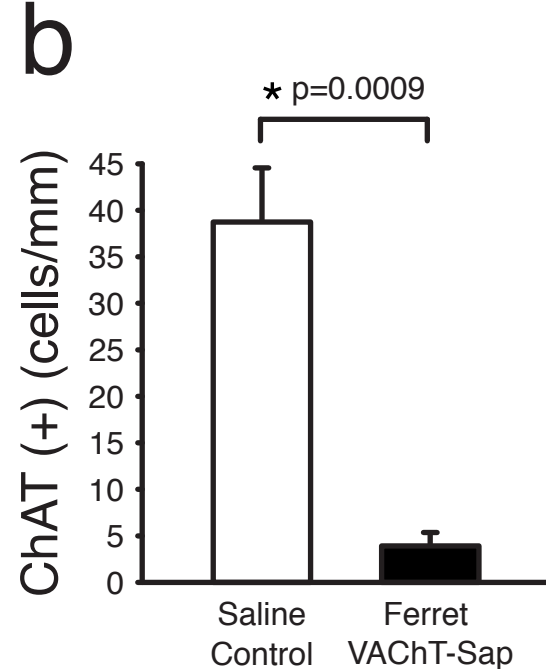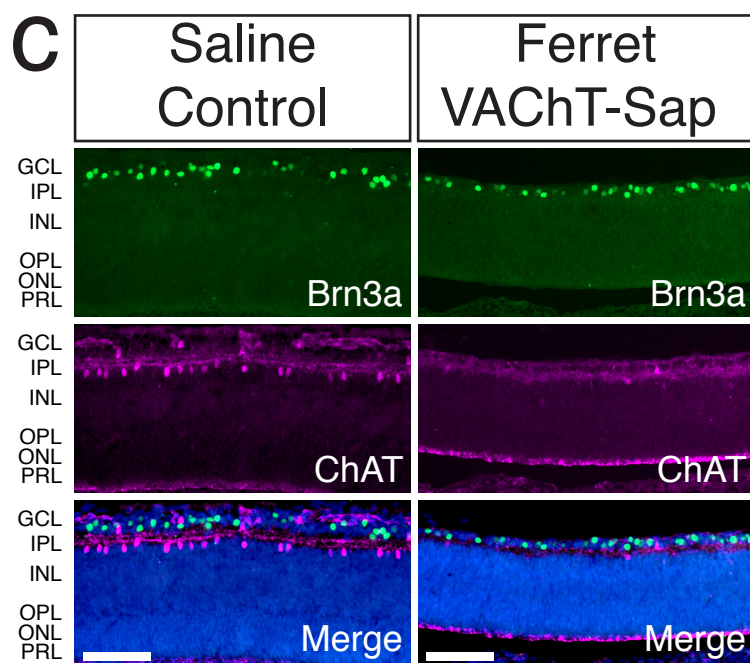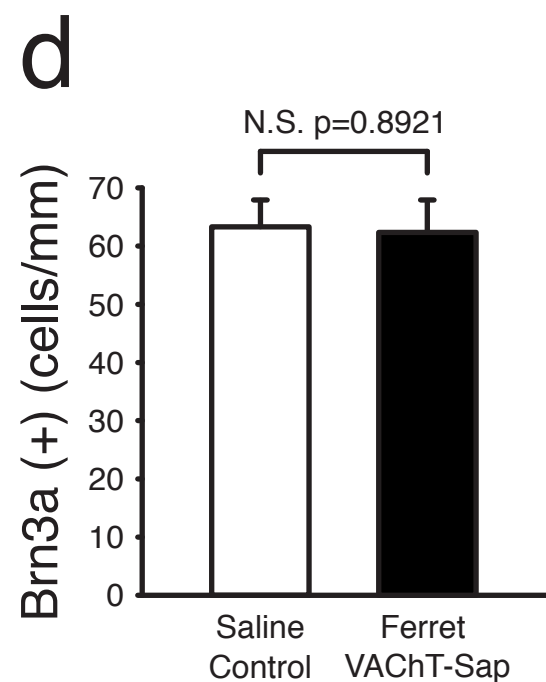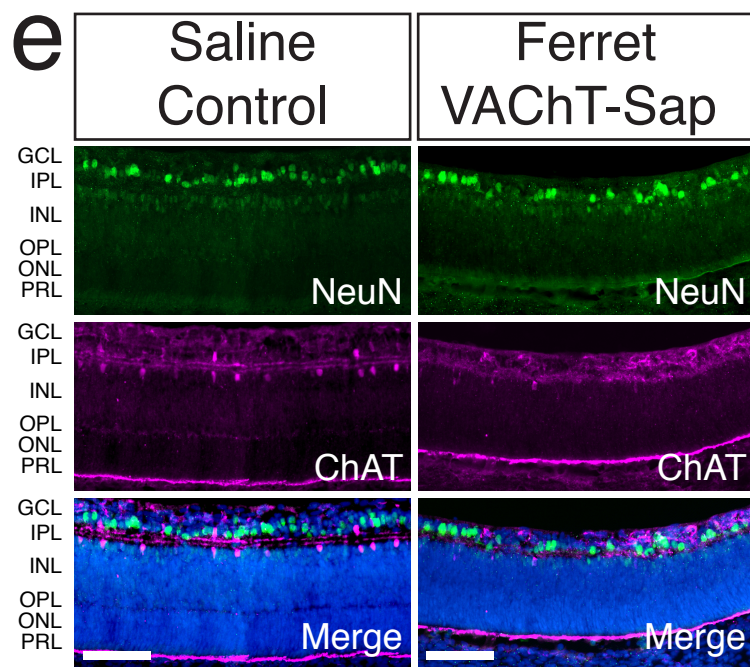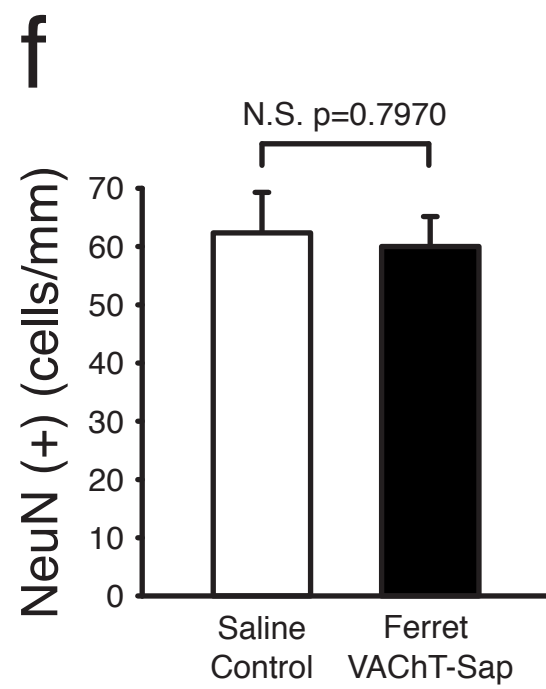

Supplement: Supplementary file 2 — Additional file 2: Ferret VAChT-Sap treatment does not ablate retinal ganglion cells. Starburst amacrine cells (SACs) and their dendrites (magenta) are immunopositive for choline acetyltransferase (ChAT). Ferret-specific vesicular acetylcholine transport protein antibody (Ferret VAChT) labels SAC dendrites (green) in the inner plexiform layer (IPL) shown here at postnatal day 10 (P10) (a). Treatment with Ferret VAChT-Sap leads to ablation of SACs and a loss of staining for SAC dendrites (a,b). Staining for Brn3a and NeuN (green) labels retinal ganglion cells (RGCs), which are not ablated by Ferret VAChT-Sap treatment (c-f). Scale bars are 100 μm. Quantification shows mean + SEM; counts were made from three saline control retinae and four Ferret VAChT-Sap-treated retinae. Statistics reflect two-tailed P values calculated from independent two sample Student’s t-tests. In a-f, ‘Merge’ panels show counterstaining with DAPI (blue) to reveal retinal cytoarchitecture. GCL, ganglion cell layer; INL, inner nuclear layer; IPL, inner plexiform layer; NS, not significant; ONL, outer nuclear layer; OPL, outer plexiform layer; PRL, photoreceptor layer. (PDF 6 MB) [file 13064_2014_271_MOESM2_ESM.pdf]

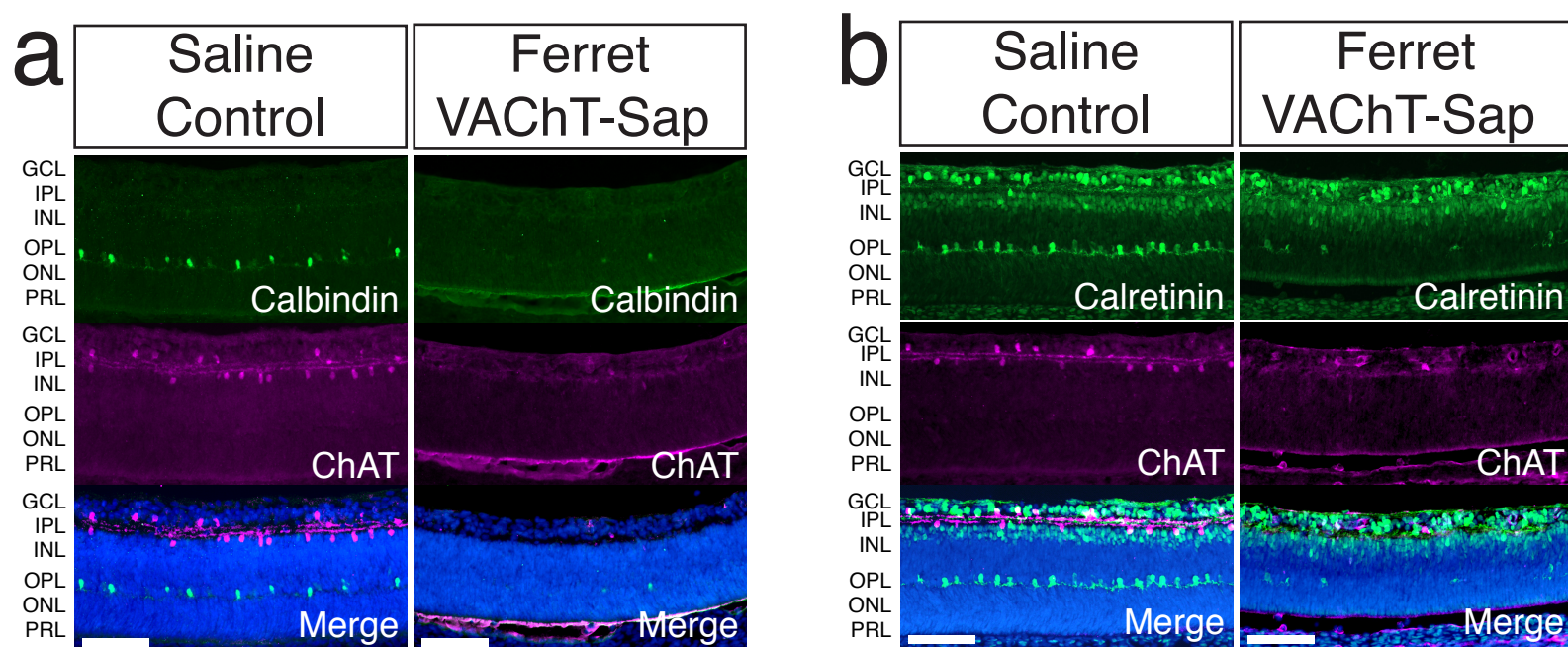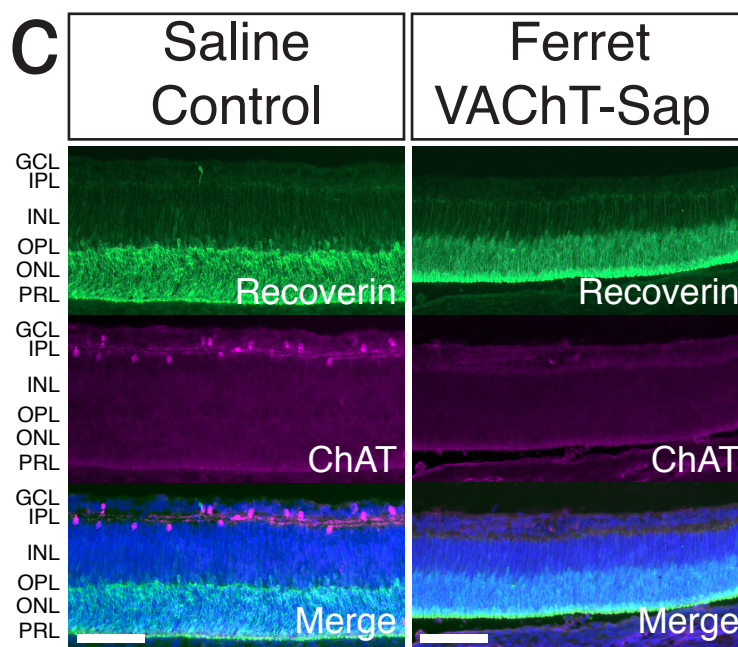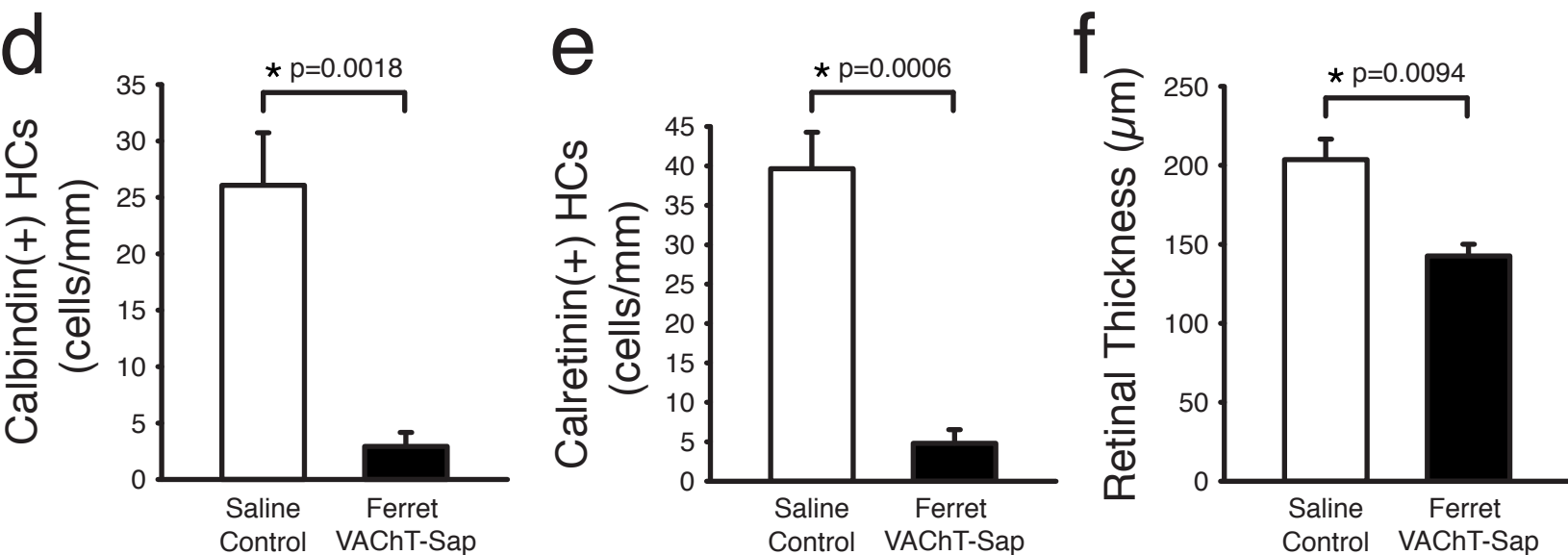

Supplement: Supplementary file 3 — Additional file 3: Ferret VAChT-Sap treatment ablates horizontal cells and decreases overall retinal thickness. Calbindin labels horizontal cells (HCs, green) in the inner nuclear layer (INL), as well as their dendrites in the outer plexiform layer (OPL) at postnatal day 10 (P10) (a). HCs are significantly ablated by Ferret VAChT-Sap treatment (a,d). Calretinin also labels HCs as well as a heterogeneous population of neurons in the ganglion cell layer (GCL) (b, green images). Ferret VAChT-Sap treatment leads to loss of calretinin (+) cells in the INL (b,e; calretinin (+) cells in the GCL were not quantified). Recoverin labels photoreceptor somas and outer segments in the outer nuclear layer (ONL) and photoreceptor layer (PRL) respectively, as well as axonal processes of some bipolar cells terminating in the inner plexiform layer (IPL) (c, green images). Ferret VAChT-Sap-treated retinae show qualitatively similar recoverin (+) labeling (c). The absolute thickness of the retinal sheet in Ferret VAChT-Sap-treated retinae is reduced relative to saline controls (f). Scale bars are 100 μm. Quantification shows mean ± SEM; counts were made from three saline control retinae and four Ferret VAChT-Sap-treated retinae. Statistics reflect two-tailed P values calculated from independent two sample Student’s t-tests. In a-c, ‘Merge’ panels show counterstaining with DAPI (blue) to reveal retinal cytoarchitecture. (PDF 5 MB) [file 13064_2014_271_MOESM3_ESM.pdf]
