# Supplementary material for: Eye-specific retinogeniculate segregation proceeds normally following disruption of patterned spontaneous retinal activity
Source: Neural Dev. 2014 Nov 7;9:25. doi: 10.1186/1749-8104-9-25 (PMC4289266; doi:10.1186/1749-8104-9-25)
Supplement: Supplementary file 4 — Additional file 4: Effects of Ferret VAChT-Sap treatment on retinal cell loss. Supplemental results word document. (DOCX 30 KB) [file 13064_2014_271_MOESM4_ESM.docx]

Additional file 4

Supplemental Results

Effects of Ferret VAChT-Sap treatment on retinal cell loss

To ablate starburst amacrine cells (SACs), we first generated a custom antibody against the C-terminal domain of the ferret vesicular acetylcholine transporter (Ferret anti-VAChT). This antibody labels SAC dendrites in the inner plexiform layer where it colocalizes with staining for choline acetyltransferase (ChAT) (Supplemental Figure 2a). We conjugated this antibody to saporin toxin to create a SAC-specific immunotoxin, Ferret VAChT-Sap (see Methods and [1]). Treatment with Ferret VAChT-Sap ablated SACs as evidenced by a loss of ChAT (+) cell bodies as well as a loss of VAChT staining in the retina at P10 (Supplemental Figure 2a). SAC density was 38.87 ± 5.79 cells/mm in saline controls (mean ± S.E.M.) and was reduced to 4.31 ± 1.07 cells/mm in Ferret VAChT-Sap-treated retinae (mean ± S.E.M.), an 88.9% ablation (Supplemental Figure 2b).

To examine the specificity of Ferret VAChT-Sap for SACs, we immunolabeled retinae with antibody markers of different retinal neuron subtypes. To assess retinal ganglion cell (RGC) populations, we employed two markers, Brn3a (Supplemental Figure 2c-d) and NeuN (Supplemental Figures 1a-c and 2e-f). Ferret VAChT-Sap treatment did not result in significant loss or alteration of the staining pattern of either of these two markers of RGCs. In retinal cryosections, saline controls had a density of 63.88 ± 4.34 Brn3a (+) cells/mm (mean ± S.E.M.) while Ferret VAChT-Sap-treated retinae contained 62.87 ± 5.14 Brn3a (+) cells/mm (mean ± S.E.M.) (Supplemental Figure 2d). Saline control retinae contained 62.67 ± 6.82 NeuN (+) cells/mm (mean ± S.E.M.) while Ferret VAChT-Sap-treated retinae contained an average of 60.4 ± 4.74 NeuN (+) cells/mm (mean ± S.E.M.) (Supplemental Figure 2f). Given that RGCs expressing Brn3a project to image forming visual targets in the brain [2], it is significant that there were no adverse effects on this population that could account for potential deficits in retinofugal projection patterns.

Intraocular injection of Ferret VAChT-Sap reduced calbindin (+) A-type horizontal cells (HCs) from 26.33 ± 4.53 in saline controls (mean cells/mm ± S.E.M.) to 3.23 ± 0.95 (mean cells/mm ± S.E.M.) in treated retinae, an 87.7% reduction (Supplemental Figure 3a and d). A separate marker of both A and B type HCs, calretinin, confirmed that Ferret VAChT-Sap treatment reduced total HC density from 39.79 ± 4.38 cells (mean/mm ± S.E.M.) in saline controls to a density of 5.06 ± 1.54 cells/mm (mean ± S.E.M.), an 87.3% reduction (Supplemental Figure 3b and e). Staining of outer retinal neurons with antibodies for recoverin revealed qualitatively similar staining patterns in control and Ferret VAChT-Sap-treated retinae (Supplemental Figure 3f).

Following Ferret VAChT-Sap treatment the absolute thickness of the retina counterstained with DAPI was significantly reduced in treated (143.74 ± 7.18 µm; mean ± S.E.M.) vs. control (204.56 ± 11.8 µm; mean ± S.E.M.) groups (Supplemental Figure 3f). This is likely primarily due to the ablation of both SACs and HCs, but may also include other cell types that were not investigated in this study. Although horizontal cells were ablated by Ferret VAChT-Sap treatment these cells are unlikely to contribute to spontaneous retinal activity due to the immaturity of photoreceptors and bipolar cell circuits at the ages we studied [3-5]. However, it is possible that secondary effects of HC/SAC ablation impact retinal waves, perhaps by perturbing molecular signaling mechanisms in the retina independent of synaptic activity.

1. Speer CM, Sun C, Chapman B: **Activity-dependent disruption of intersublaminar spaces and ABAKAN expression does not impact functional on and off organization in the ferret retinogeniculate system.** *Neural Dev* 2011, **6:**7.

2. Quina LA, Pak W, Lanier J, Banwait P, Gratwick K, Liu Y, Velasquez T, O'Leary DD, Goulding M, Turner EE: **Brn3a-expressing retinal ganglion cells project specifically to thalamocortical and collicular visual pathways.** *J Neurosci* 2005, **25:**11595-11604.

3. Miller ED, Tran MN, Wong GK, Oakley DM, Wong RO: **Morphological differentiation of bipolar cells in the ferret retina.** *Vis Neurosci* 1999, **16:**1133-1144.

4. Johnson PT, Williams RR, Reese BE: **Developmental patterns of protein expression in photoreceptors implicate distinct environmental versus cell-intrinsic mechanisms.** *Vis Neurosci* 2001, **18:**157-168.

5. Greiner JV, Weidman TA: **Histogenesis of the ferret retina.** *Exp Eye Res* 1981, **33:**315-332.
